# Statistical Techniques Complement UML When Developing Domain Models of Complex Dynamical Biosystems

**DOI:** 10.1371/journal.pone.0160834

**Published:** 2016-08-29

**Authors:** Richard A. Williams, Jon Timmis, Eva E. Qwarnstrom

**Affiliations:** 1 Department of Computer Science, University of York, York, United Kingdom; 2 York Computational Immunology Laboratory, University of York, York, United Kingdom; 3 Department of Electronics, University of York, York, United Kingdom; 4 Department of Infection, Immunity and Cardiovascular Disease, Medical School, University of Sheffield, Sheffield, United Kingdom; 5 Affiliated, Department of Pathology, School of Medicine, University of Washington, Seattle, Washington, United States of America; Consiglio Nazionale delle Ricerche, ITALY

## Abstract

Computational modelling and simulation is increasingly being used to complement traditional wet-lab techniques when investigating the mechanistic behaviours of complex biological systems. In order to ensure computational models are fit for purpose, it is essential that the abstracted view of biology captured in the computational model, is clearly and unambiguously defined within a conceptual model of the biological domain (a *domain model*), that acts to accurately represent the biological system and to document the functional requirements for the resultant computational model. We present a domain model of the IL-1 stimulated NF-κB signalling pathway, which unambiguously defines the spatial, temporal and stochastic requirements for our future computational model. Through the development of this model, we observe that, in isolation, UML is not sufficient for the purpose of creating a domain model, and that a number of descriptive and multivariate statistical techniques provide complementary perspectives, in particular when modelling the heterogeneity of dynamics at the single-cell level. We believe this approach of using UML to define the structure and interactions within a complex system, along with statistics to define the stochastic and dynamic nature of complex systems, is crucial for ensuring that conceptual models of complex dynamical biosystems, which are developed using UML, are fit for purpose, and unambiguously define the functional requirements for the resultant computational model.

## Introduction

**Fig 1:**
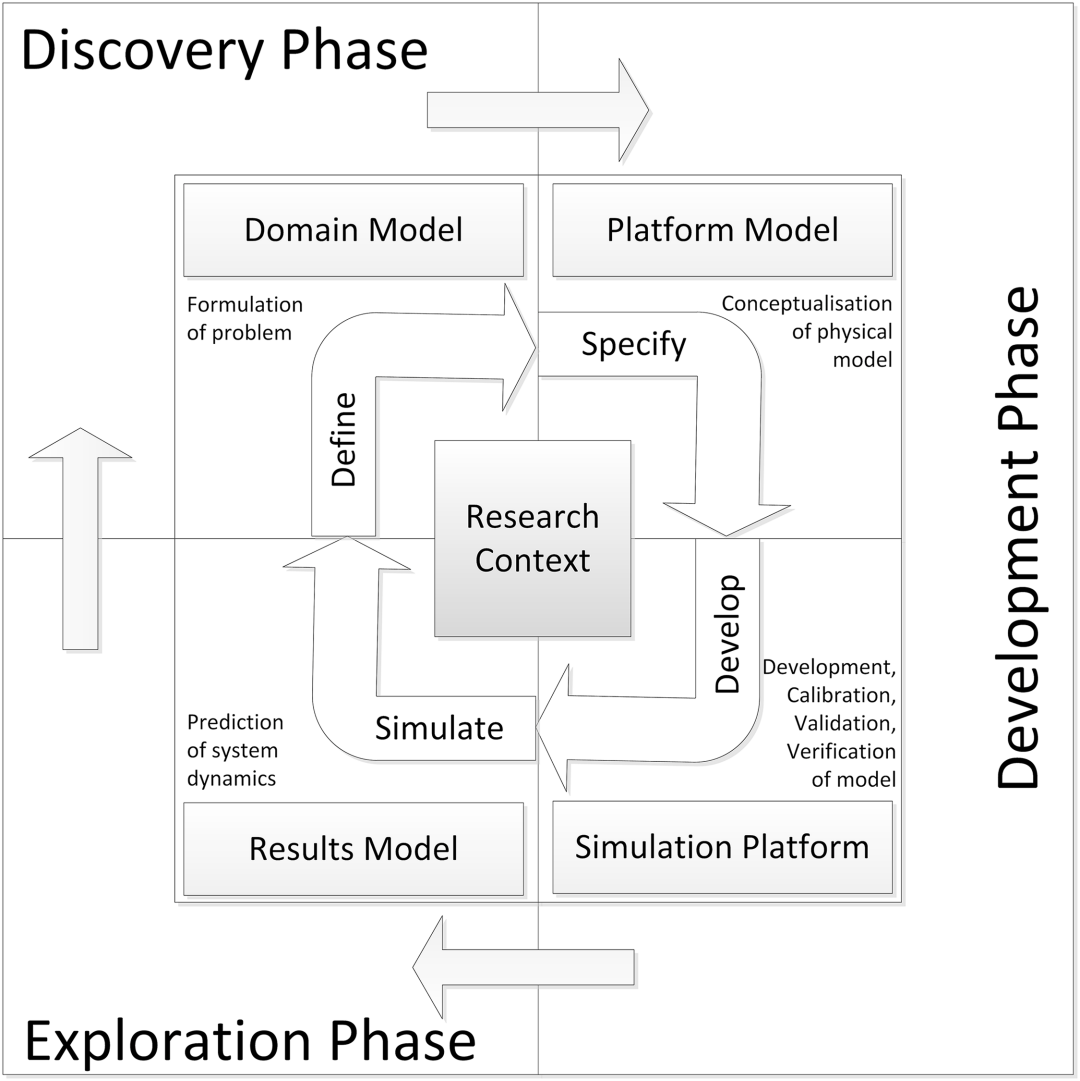
The CoSMoS Process. The CoSMoS process advocates an iterative lifecycle, consisting of three separate phases (discovery, development and exploration), and creation of four key project artefacts (domain model, platform model, simulation platform, and results model). The discovery phase focuses on formulation of the problems to be investigated through use of the computational model, resulting in creation of a functional specification of the required biological behaviour to be simulated (domain model). The development phase focuses on transforming the domain model into a technical specification (platform model) specific to the programming language(s) and computer architectures to be used, and actual development of the computational model (simulation platform), including calibration, validation and verification. The exploration phase focuses on the *in silico* experimentation to investigate the biological problems of interest, and the generation of predictions (documented in the results model), which facilitate the generation of novel hypotheses for subsequent testing in the biological arena.

Over the past twenty years, a systems approach to research has become more widespread within biology. Researchers in the biological sciences are now increasingly using computer models and simulations to better understand intercellular and intracellular processes of living organisms. The merits of a systems biology approach have been discussed in depth by Kitano [1–3], with his project lifecycle diagram (Fig 1 from [2]) having the potential to become the classic diagrammatic representation of how systems biology is underpinned by a hypothesis-driven research cycle. More recently, the Complex Systems Modelling and Simulation (CoSMoS) process has been developed [4–6]. This process provides a framework of leading practice for developing and using simulations to explore complex systems, and is comparable to project lifecycle methodologies used in industry. Like these traditional methodologies, the CoSMoS process is organised around phases, which contain a set of products (deliverables), and associated activities. The CoSMoS process has three phases: Discovery phase, which establishes the scientific basis of the project, identifies and models the domain of interest, and formulates scientific questions; the Development phase, which produces the actual simulator; and the Exploration phase, which uses the simulator for *in silico* experimentation; the results of which are used to explore the scientific questions defined previously. Along with these phases, there are key products associated with CoSMoS projects: Domain Model, Platform Model, Simulation Platform, and the Results Model (see Fig 1).

**Fig 2:**
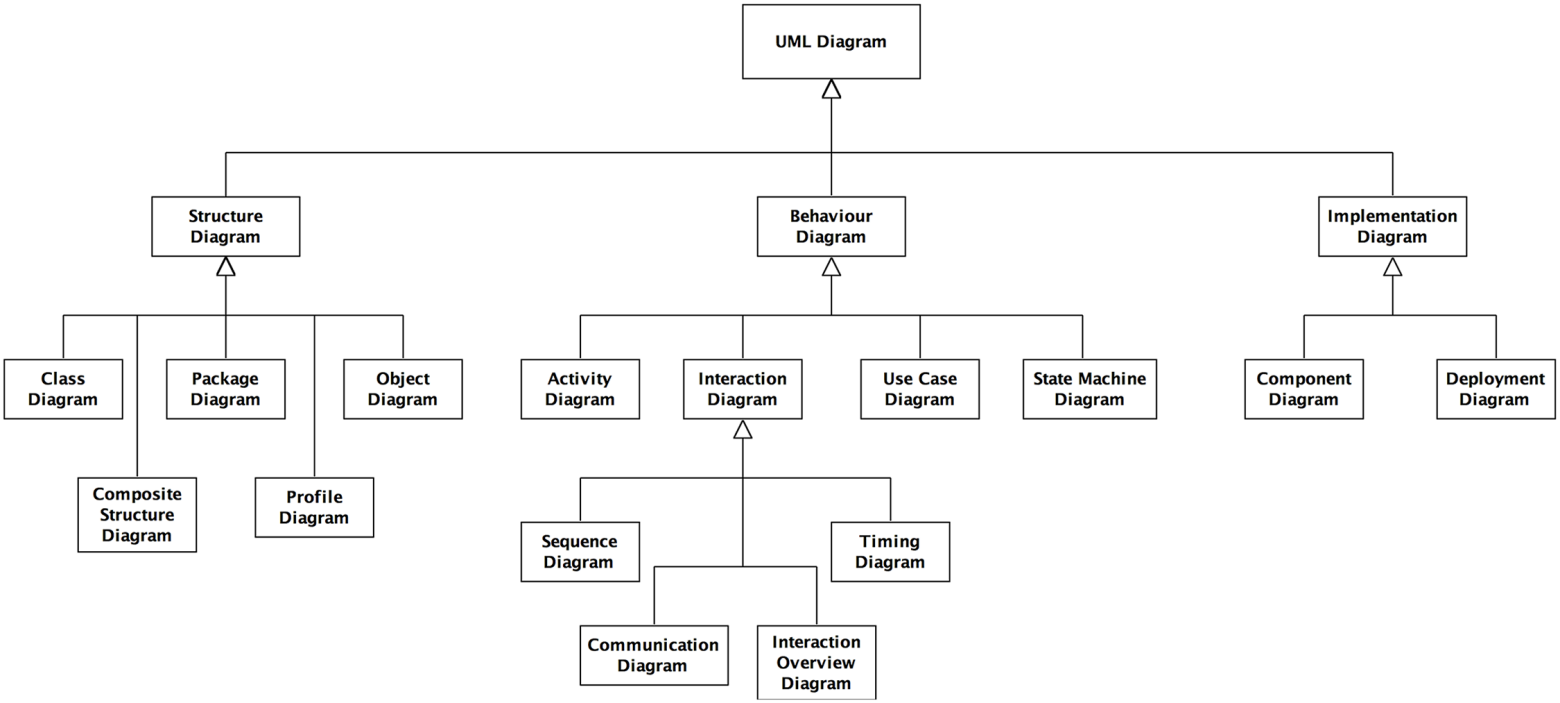
Taxonomy of UML diagramming notations. Structure diagrams show the static structure of the components within a system, and comprise: Class, Composite Structure, Package, Profile and Object diagrams. Behaviour diagrams show the dynamic behaviour of the components within a system, and comprise: Activity, Sequence, Communication, State Machine, Use Case, Interaction Overview, and Timing diagrams. Finally, implementation diagrams comprise Component and Deployment diagrams (after [10]).

The domain model is an abstract representation of the actual system of interest (the Domain), which documents our understanding of the domain into explicit statements, that may relate to assumptions, constraints, definitions, and indeed relationships or interactions between components of the domain (discussed by Polack et al [7, 8]). One approach to semi-formally document the required functionality of a system, which has become the *de facto* standard for modelling software systems by the software engineering community [9], uses the Unified Modelling Language (UML). The UML specification (version 2.4) [10] defines 14 separate diagramming notations, split across three main groups: structure diagrams, which show the static structure of components within a system; behaviour diagrams, which show the dynamic behaviour(s) of components within a system; and implementation diagrams, which show the hardware and software infrastructures within a system (see Fig 2 for the taxonomy of UML diagramming notations).

With respect to biology, UML has previously been used by Bersini et al [11] to diagrammatically model the content and functions associated with biological systems, and [12] to model Rosen’s Metabolism-Replacement system that is used within relational biology. With specific reference to domain modelling, UML has also been used by Read et al [13], to define the domain model of the intercellular interactions within an autoimmune disease (Experimental Autoimmune Encephalomyelitis, an animal form of multiple sclerosis), and Alden et al [4], to define the domain model of cell interactions within tissue formation in the immune system (specifically the lymphoid organ). In addition to UML, the Systems Biology Graphical Notation (SBGN) [14] has also been used as a diagrammatic notation to model biological systems, such as the Toll-like receptor network [15] and the mTOR signalling network [16]. The SBGN was developed by an international collaboration of biochemists, computational biologists and computer scientists, with the overriding objective to allow scientists to diagrammatically represent networks of biochemical interactions using standardised terminology and notation. Whereas UML contains 14 different notations, the SBGN currently only contains 3, being: the process diagram, the entity-relationship diagram, and the activity diagram.

Taking these 3 notations in turn, firstly, the process diagrams are used for modelling the interactions that take place between biomolecules and the various state-transitions that occur as part of the biochemical reaction. They are able to convey the temporal aspects of molecular events occurring in biochemical reactions, and are analogous to UML sequence and communication diagrams. The main drawback with process diagrams appears to be that a given component must appear multiple times on the same diagram if it exists under several states, whereas in UML you can have one entity with several activities coming off, that through the use of *guard conditions*, can specify which activity occurs under specific circumstances. Indeed, the requirement for SBGN process diagrams to diagrammatically define all states that a component can take, can become problematic. For example, a biological component that acts as a *hub* in a network will have a large number of connections and therefore possible network states. These all have to be defined separately in process diagrams, which leads to the issue of *combinatorial explosion* identified by [17]. Secondly, the entity-relationship diagrams are based on Kohn’s molecular interaction maps and are used for modelling the relationships between biomolecules. These focus on the influences that entities have on each other, but not the state transformations that occur following interactions; they are akin to UML class diagrams and activity diagrams. Unlike the process diagrams, an entity appears only once, which is closer to the approach of UML. An enhancement over UML with respect to modelling biology is that these diagrams have specific notations for low-level biochemical reactions such as phosphorylation, which can be displayed on specific amino acid residues of protein entities. Finally, the activity flow diagrams are used for modelling the activities of biomolecules at a high-level of abstraction. They can be used to convey component-level interactions (e.g. protein-protein), without the need to show the detail of specific chemical reactions at the level of individual amino acids (e.g. phosphorylation events). As the activity diagram ignores the specific biochemical processes that entities are involved in, and their associated state transitions, they are quite compact in nature, and can be thought of as the typical network diagram found in traditional biochemical textbooks. As per UML, these 3 notations complement each other and are used to diagrammatically model different aspects/views of the biological system.

With respect to the conceptual modelling of complex biological systems, we agree with Grizzi [18] who advise that complex systems can be viewed from many perspectives, and therefore can be described in many ways, each of which will be only partially true. We believe that SBGN and UML are both suitable for developing domain models of complex biological systems, however we do not believe that SBGN will be suitable for the platform model (technical specification) that will be developed in the next phase of our CoSMoS project. As such, we have selected UML as the technique of choice, so that we are able to utilise a single diagrammatic notation throughout the full lifecycle of our CoSMoS project.

### The Domain

The NF-*κ*B signalling pathway is one of the key signalling pathways involved in the control and regulation of the immune system [19]. Activation of the NF-*κ*B transcription factor and signalling pathway is a tightly regulated event, involving activation of a number of signalling components [20]. NF-*κ*B is normally sequestered in the cytosol of non-stimulated cells and consequently must be translocated into the nucleus to function as a transcriptional activator of target genes. NF-*κ*B is activated by a wide variety of different extracellular stimuli, including proinflammatory signalling molecules, bacteria, viruses, and physical and chemical stresses [21].

As previously advocated by Kitano [2], we also believe that computational modelling and simulation can complement wet-lab experimental approaches. With specific reference to the NF-*κ*B intracellular signalling pathway, there is potential to facilitate a more comprehensive understanding of the underlying mechanistic behaviours of the system, which could then be harnessed for identifying targets for therapeutic interventions to resolve system dysregulation [1]. Existing Ordinary Differential Equation (ODE) based models [22–24] have been useful in increasing our understanding at the cell population-level, however we believe that the field will gain further benefits from computational models at the single-cell level that contain increased scope and granularity of components over and above these mathematical models, and will also allow us to investigate the mechanistic underpinning (i.e. not just the dynamics) of the system (see [25] and our recent review [26]). Our long term objective is to build on previous work [27, 28] that used agent-based modelling, and develop a detailed model of the IL-1 stimulated NF-*κ*B signalling pathway, for the purpose of performing *in silico* experimentation as a basis of hypothesis generation for the biological domain.

In order to ensure that the computational model appropriately reflects the biological case-study, good software engineering practices through a principled approach to design and development, such as the CoSMoS process [5] should be adopted. This advocates the creation of a *domain model*, which captures the essential processes and entities of the real-world system under study; in particular, the emergent behaviour, at an appropriate level of abstraction. Having a separate domain model (akin to a functional specification) from a *platform model* (which details how the simulation is designed, and is akin to a technical specification), allows for the concentration on biological fact and for us to scope the system to be modelled, and therefore not be biased by implementation specific details at this early stage of the research project.

There exist substantial quantities of literature on the NF-*κ*B signalling pathway, with various aspects of the pathway being independently studied by a wide variety of labs. Furthermore, it is generally understood that representing every aspect of a real-world system in models and simulations is computationally intractable, and therefore requires focus on a subset of the properties and behaviours for subsequent model-driven investigations. One of the primary purposes of the domain model is to capture this subset of real-world system properties, and therefore provide a definition of the abstraction level taken for the modelling project. This paper presents our domain model of the IL-1 stimulated NF-*κ*B signalling pathway, based on the previously published work of Carlotti et al [29, 30] and Yang et al [31, 32], who used fluorescent protein constructs and confocal fluorescence microscopy to investigate pathway dynamics in living cells. Data from the observations presented in the publications by Yang et al were selected for analysis to test the effectiveness in using the Unified Modelling Language in developing a domain model of the widely distributed data.

## Materials and Methods

The domain model utilises the empirical findings of Carlotti et al [29, 30] and Yang et al [31, 32], with the statistical analysis being specifically based on a subset of data from Yang et al [32]. This subset contained measurements from single-cell analysis performed on 88 cells: 52 were transfected with I*κ*B*α* Enhanced Green Fluorescent Protein (EGFP) and stimulated with IL-1; and 36 were transfected with I*κ*B*α*-EGFP, but not stimulated with extracellular ligand, thus representing a *control* group. Single-cell analysis on live cells, include continuous monitoring of the same set of cells over time. All measurements within the data related to cytoplasmic fluorescence and were taken over a period of one hour, at intervals corresponding to 0, 10, 30 and 60 min. The subset of data used within the statistical analysis of this manuscript can be found in S1 and S2 Tables of the supplementary information. The data sets were divided into 3 groups based on transfection levels of the exogenous protein as in the original analysis by Yang et al: 0-1.5 fluorescent units (corresponding to up to 4 fold levels of the endogenous protein); 1.5-3.0 fluorescent units (4-8 fold levels of the endogenous protein); and above 3.0 fluorescent units (above 8 fold levels of the endogenous protein) [32].

The domain model was developed in an iterative manner by the modeller (RAW), senior software engineer (JT) and domain expert (EEQ), using the deep-curation approach [33]. We have chosen to follow the approach of Read et al [13] in using UML as the basis to semi-formally define the domain model of our complex dynamical biosystem. Along with a number of UML diagrammatic notations (UML v2.4, [10]), a number of less formal cartoon diagrams were also used to ensure the biological meaning could be conveyed efficiently. Furthermore, a number of statistical techniques were used to complement UML when modelling the temporal dynamics and stochastic characteristics of the system. Initial focus is the emergent system-wide behaviours of the pathway, before increasing the level of detail to the interactions between system components, and then the dynamics of individual components. The domain model is presented in a top-down manner, comprising three levels of abstraction, as defined below:

1. **A system-level overview of the domain model**. This highly abstract level provides an outline of the biology of the IL-1 stimulated NF-*κ*B signalling pathway. Particular focus is made to the behaviours of the system following induction by extracellular signal, and how these are believed to correspond to phenomena observed in the real-world domain. This abstraction level of the domain model does not make use of UML, but instead utilises less formal cartoon diagrams to convey system-wide properties, along with a number of statistical approaches to convey temporal dynamics. In particular, we have used the R data analysis and graphics software [34] to perform Chi-squared goodness of fit tests to ascertain the statistical distribution of the wet-lab data, along with hierarchical clustering and Principal Component Analysis to investigate any underlying groupings with the dataset of Yang et al [32] (see supplementary information for detailed descriptions).
2. **Modelling component-level interactions of the domain model**. This medium level abstraction, decomposes the IL-1 stimulated NF-*κ*B signalling pathway into its constituent molecular components. This level models an abstracted view of the key molecular interactions between the components, that together give rise to the emergent behaviours of the system. A cartoon diagram, along with UML communication and activity diagrams have been used in modelling these component-level interactions.
3. **Modelling individual component dynamics**. This level of abstraction provides the greatest detail within the domain model, through modelling the dynamics of individual components within the system. A set of linked UML state machine diagrams have been used to develop this level of the model.

### Validation of the Domain Model

The iterative approach to developing the domain model within a CoSMoS project provides an ability to use a number of validation techniques during model formulation. Balci [35] has extensively reviewed the verification and validation techniques that are suitable for computational model development and simulation-based experiments. We have used a subset of these techniques to validate our domain model, comprising: audits by the senior software engineer to ensure that the modelling adheres to established practices; desk checking by the modeller to ensure that individual diagrammatic and statistical models are correct, complete, consistent and unambiguous; face validation by the domain expert to compare the complete domain model against her detailed understanding and judgment of the real-world biological system; and structured walkthroughs by the whole group (modeller, senior software engineer and domain expert) to detect and document faults.

## Results

As recently argued by Read et al [36], the domain model defines our understanding of how system-level behaviours emerge from the cumulative actions of lower-level components, such as intracellular signalling molecules. The domain model presented in this paper represents the subset of signalling components that give rise to system-level dynamics. It was developed in a top-down manner, through close alignment to the process advocated by Read et al, and comprises three levels of abstraction, at the system-level, component-level, and individual components.

### Modelling System-Level Properties

This highly abstract level provides an outline of the biology of the IL-1 stimulated NF-*κ*B signalling pathway. Particular focus is made to the behaviours of the system following induction by extracellular signal, and how these are believed to correspond to phenomena observed in the real-world domain.

#### Modelling Expected Behaviours

**Fig 3:**
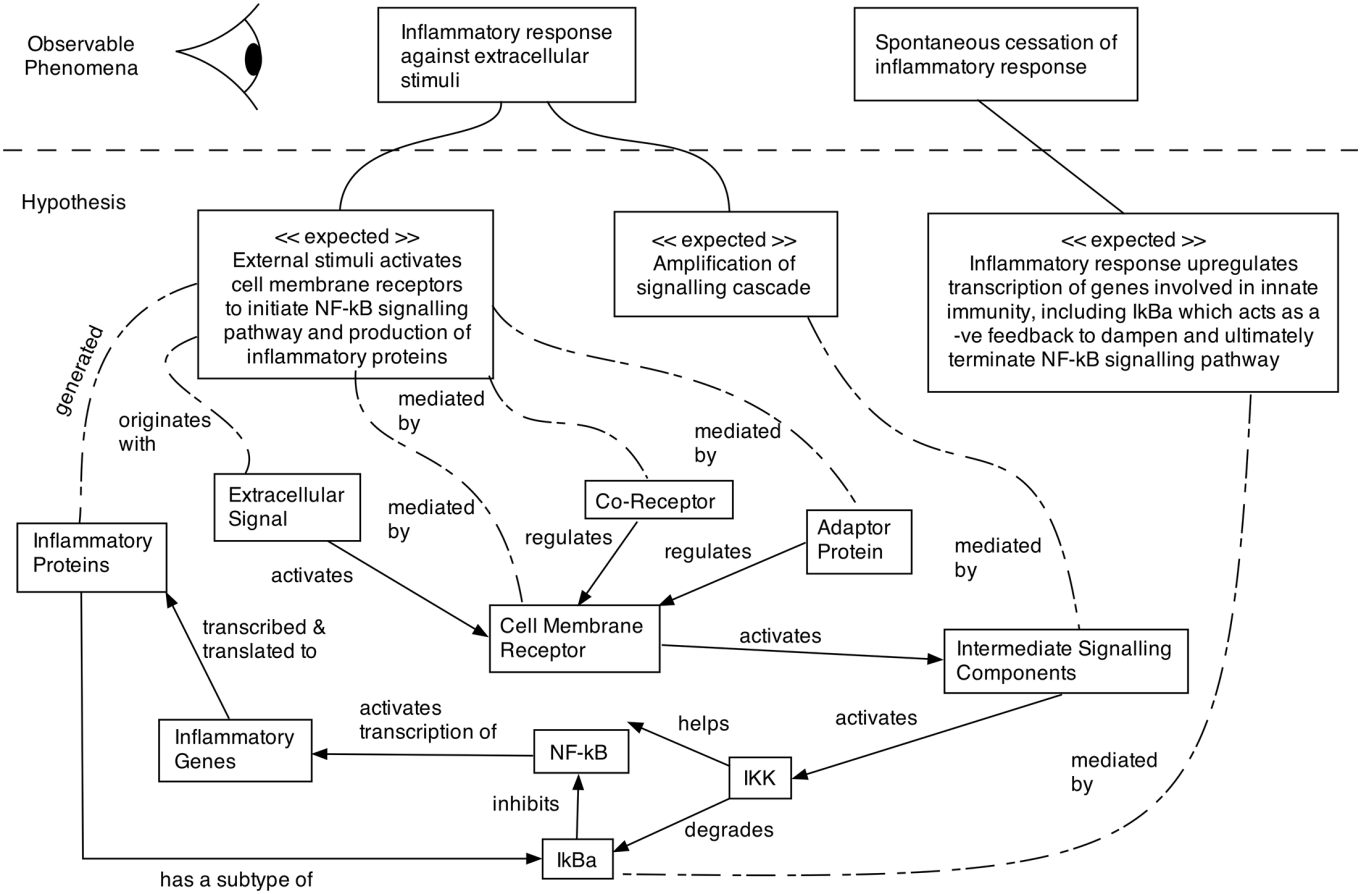
Expected behaviours diagram. Expected behaviours diagram depicting the observable phenomena of the IL-1 stimulated NF-*κ*B signalling pathway; the behaviours that are hypothesised to be responsible for these phenomena; and at an abstracted level the components of the complex system that are believed to be responsible for the development of these emergent behaviours. At the highest level of the system, activation of the NF-*κ*B pathway initiates a transitory inflammatory response (the system dynamics automatically cease the response). It is hypothesised (expected) that these phenomena occur through interaction of three functional modules that relate to activation of cell membrane receptors, amplification of the signalling cascade, and upregulation of transcription. Developed from the reviews of [37–39].

Following the approach of Andrews et al [5] and the example of Read et al [13], we have chosen to commence the domain modelling process with a cartoon-like diagram, termed an *expected behaviours* diagram (Fig 3). This diagram depicts the observable phenomena of the IL-1 stimulated NF-*κ*B signalling pathway, along with the known interactions between system components that generate system-wide behaviours. The diagram also provides us with an opportunity to define a number of hypotheses on how these known component interactions may yield the observable phenomena. The expected behaviours diagram therefore provides a diagrammatic view of the relationship between the real-world domain and the domain model [13].

The top section of Fig 3 defines the observable phenomena of the signalling pathway, in that the pathway results in an inflammatory response against extracellular stimuli, and that after a period of time, this inflammatory response ceases. The dotted horizontal line demarcates these observable phenomena from hypotheses that are believed to be responsible for their emergence. These hypotheses consist of expected behaviours (using ‘<<expected>>’ tags) that emerge through the interactions of the underlying system components. The known interactions between system components are represented through a set of solid, directed lines, whilst the expected behaviours are linked to these system components through a set of dashed lines.

Wet-lab experimental research into NF-*κ*B since its discovery in 1986 [40], has identified that a large number of inflammatory signals (extracellular stimuli) activate cell membrane receptors to initiate its signalling pathway. Signal transduction through the intracellular network, via activation of various intermediate signalling components, amplifies the signalling cascade so that a short, transitory burst of stimuli, induces the transcription of target genes and the corresponding translation of the mRNA into proteins. One of the early genes activated by NF-*κ*B is its inhibitor I*κ*B*α*, which induces negative feedback to dampen the inflammatory response [41].

#### Modelling Physical Containment

**Fig 4:**
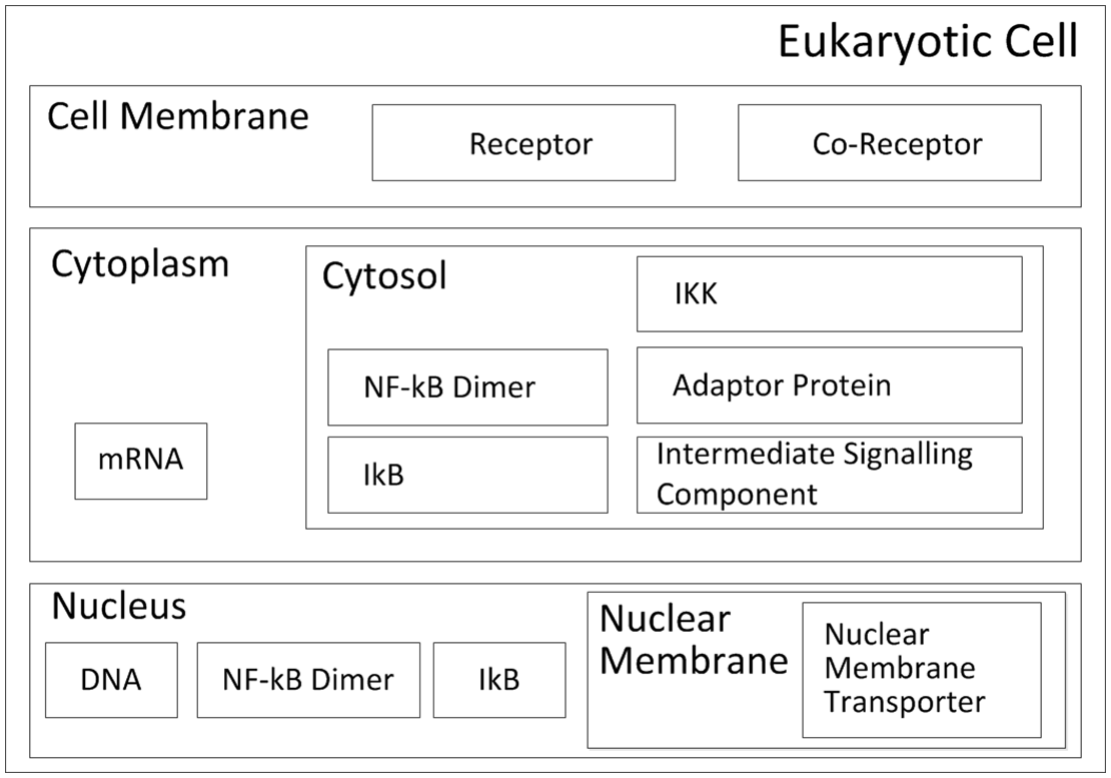
Cartoon-like containment diagram. Cartoon-like containment diagram showing the physical containment of the components involved in the IL-1 stimulated NF-*κ*B signalling pathway and the physical environment in which they are situated within a Eukaryotic cell. We believe that this is a much more intuitive way of representing physical containment than the corresponding UML class containment diagram (not shown). Developed from [42].

The spatial relationships of the components detailed within the expected behaviours diagram can be seen in the cartoon containment diagram (see Fig 4), which provides an abstract representation of a Eukaryotic cell. For the purposes of modelling the IL-1 stimulated NF-*κ*B signalling pathway, the cellular structure can be abstracted away to contain just three cellular structures: the membrane, which for our purposes contains the cell membrane receptor and co-receptor proteins; the cytoplasm, which contains the cytosol (intracellular fluid) that further contains: adaptor proteins, intermediate signalling components, NF-*κ*B, I*κ*B*α*, and the mRNA generated from gene transcription; and the nucleus, which contains DNA, its nuclear membrane, which houses the nuclear membrane transporter proteins involved in translocation (movement of proteins between cytoplasm and nucleus), and the NF-*κ*B and I*κ*B*α* that have been translocated from the cytoplasm.

#### Modelling Dynamics

The single-cell analysis work of Carlotti et al and Yang et al generated time-series fluorescence data relating to the dynamics of NF-*κ*B translocation and I*κ*B*α* degradation, which demonstrated that genetically identical cells in a standard environment display significant differences in their response to perturbations [29–32] (see S1 and S2 Tables). At a molecular level, this was demonstrated to correlate with the level of protein expression within individual cells, and to underlie the complexity and variations seen within biological populations [29–32, 43]. Unfortunately, UML does not currently have a mechanism for depicting this variation between individuals within a population. As such, we have found UML deficient in conveying the dynamics of I*κ*B*α* degradation (along with the associated NF-*κ*B release and subsequent activation), and also deficient in modelling the quantitative aspects of the signalling pathway. We have therefore used a number of statistical techniques to complement the UML and cartoon diagrams, in order to develop a more comprehensive domain model of the signalling pathway.

**Fig 5:**
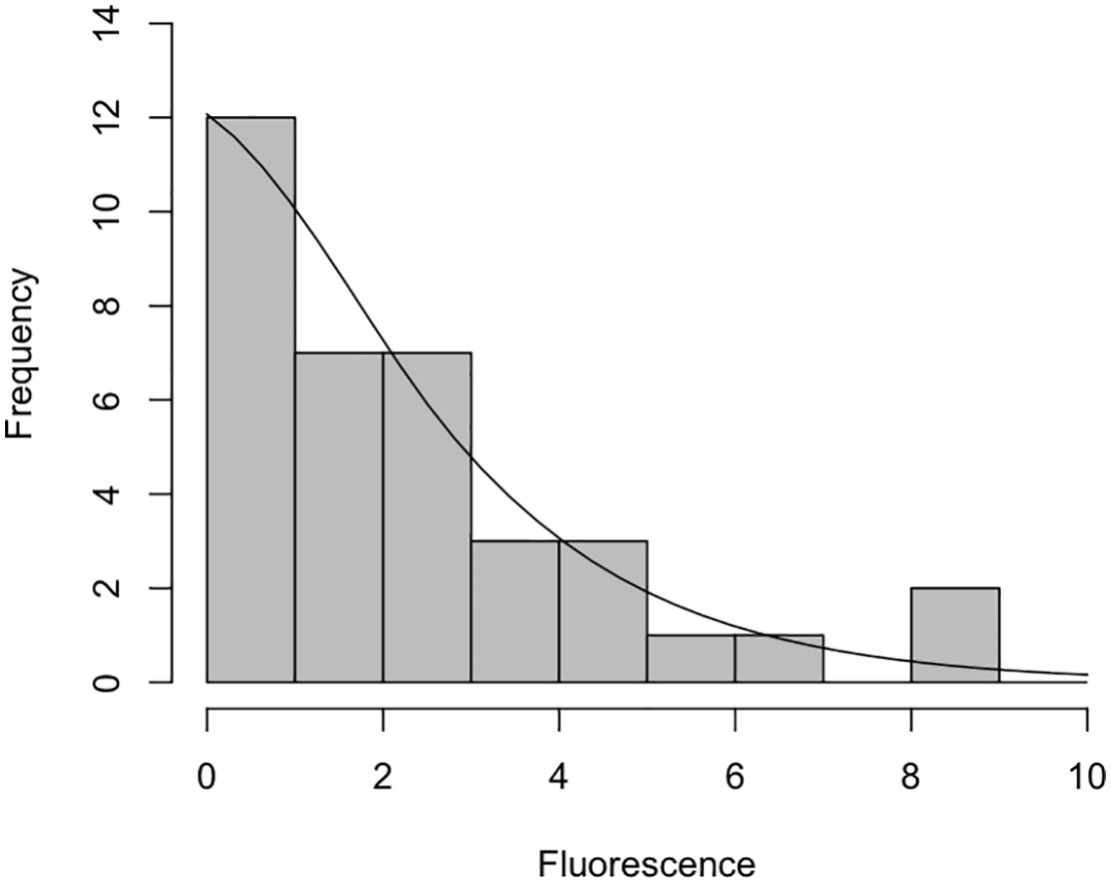
Histogram of control observations. Histrogram of control observations from the dataset of [31], that have been binned (grouped) using an integer interval of initial (time 0 min) fluorescence. The superimposed line represents a Negative Binomial distribution, using the median calculated from the raw data. The median average has been calculated as 1.947153.

**Fig 6:**
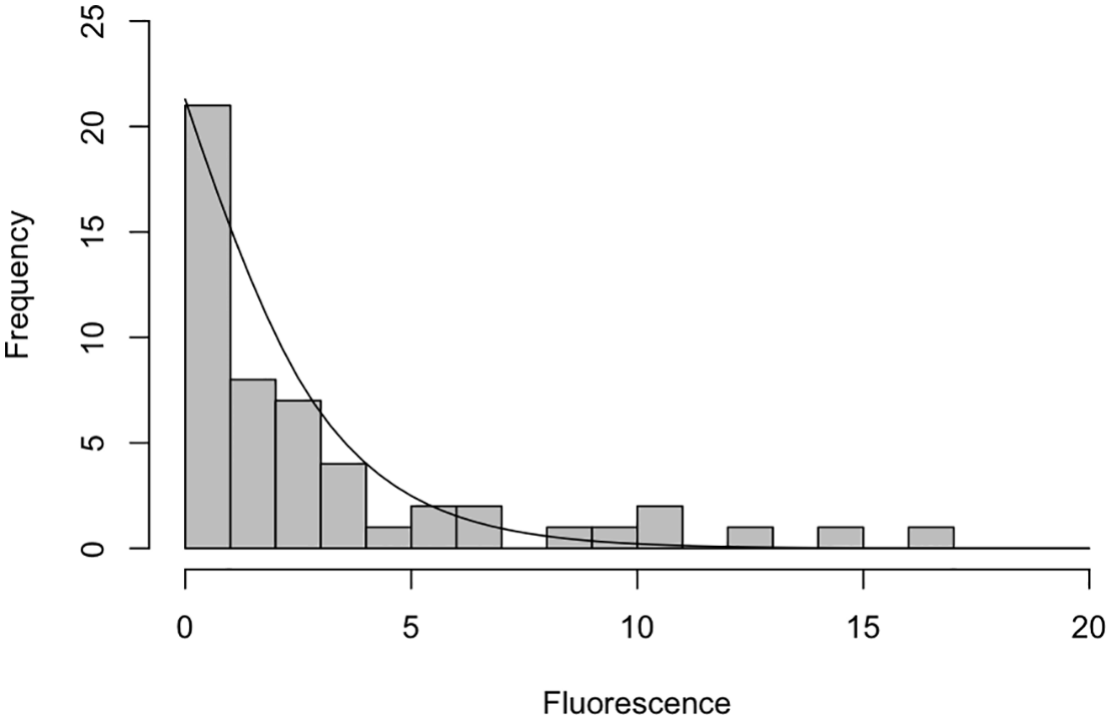
Histogram of IL-1 stimulated observations. Histogram of IL-1 stimulated observations from the dataset of [31], that have been binned (grouped) using an integer interval of initial (time 0 min) fluorescence. The superimposed line represents a Negative Binomial distribution, using the median calculated from the raw data. The median average has been calculated as 1.729876.

The general aspect of variation was later discussed by Tijskens et al [44] and Elowitz et al [45], when they conjecture that a degree of variance is inherent to all aspects of biology due to the underlying stochastic physiological events of individual cells. We feel that this should be explored further within the domain model. We have used two-tailed Chi-squared (*χ*^2^) goodness of fit tests to ascertain that the single-cell analysis (I*κ*B*α* degradation) fluorescence data approximates to a *Negative Binomial distribution*, which we believe follows the usual patterns in biology of variation due to stochasticity [46]. Figs 5 and 6 illustrate how the control and IL-1 stimulated single-cell data (at time 0 min) approximate to negative binomial distribution for the population of cells (see Supplementary Information for calculations).

**Fig 7:**
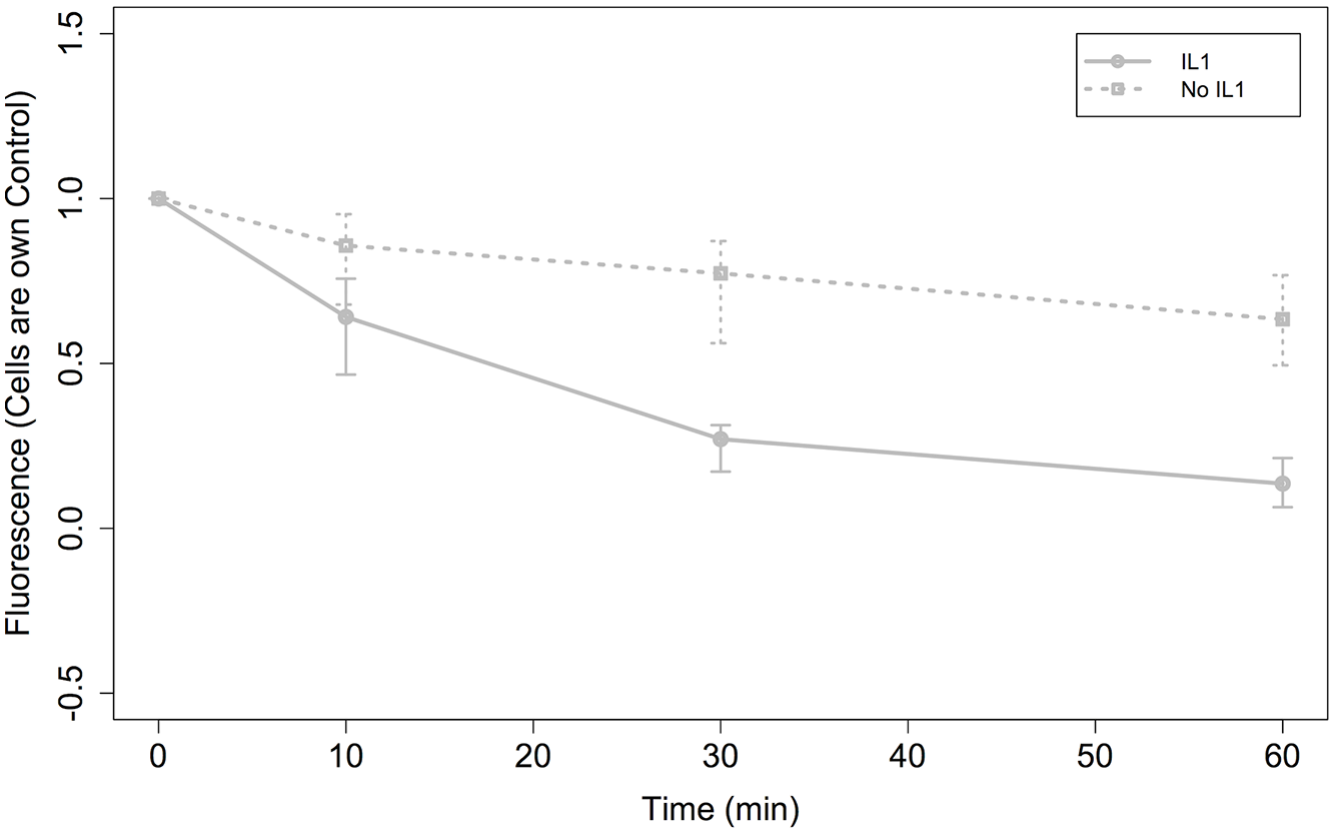
Graph of median average fluorescence. Graph of median average fluorescence for control (No IL-1) and IL-1 stimulated observations from [31]. The data has been transformed so that each cell has become its own control. The error bars illustrate the spread of observations between the 25th and 75th percentiles.

Due to the stochastic nature of the process and the cell-to-cell variation, data on I*κ*B*α* degradation by the cytokine IL-1 were expressed relative to unstimulated levels at time 0, using each cell as its own control. This is consistent with Bliss and Fisher [46], who advise that an adequate fit of data to the negative binomial distribution provides a justification for transformation of the data to stabilize the variance, as a preparatory step for further statistical analysis by other techniques. Fig 7 is a graph of the control (unstimulated) and IL-1 stimulated data using a subset of data that had initial fluorescence up to and including 1.5 arbitrary fluorescence units using median average and variance bars for interquartile ranges (25th to 75th percentiles). This accurately reproduces the findings of Yang et al, who demonstrated a pronounced reduction in I*κ*B*α* degradation levels at fluorescence levels higher than 1-1.5 [31, 32]. It can be seen that good separation is gained at 30 min onwards, with a little overlap still apparent at 10 min. The rates of degradation are 0.366 fluorescence units per hour for control and 0.864 fluorescence units per hour for IL-1 stimulated.

*Hierarchical cluster analysis* was used to find similarities in the single-cell observations and to assist us in understanding the significance of the characteristics of the groups [47, 48]. This was performed using seven different clustering algorithms (Ward, single, complete, average, McQuitty, median and centroid), which are all part of the *hclust* function within the standard R library. The resulting dendrograms for each method (not shown) were consistent in that no clear clustering was evident between unstimulated and stimulated cells (see Supplementary Information).

Further investigation used *Principal Component Analysis* (PCA), a powerful approach for ascertaining natural groupings and a common multivariate technique for exploration and reduction of high-dimensional data. It identifies underlying patterns within the data by producing linear combinations of the underlying orthogonal variables within the dataset [49]. Therefore it can be used to reduce the dimensionality of data for detecting underlying structures [50]. The variances associated with the four principal components within the data (PC1-4), indicated that only a single principal component (PC1) is required to explain the variation. In addition, it was found that separation of observations using PC1 was dominated by the fluorescence measurements at time 0, 10 and 30 min (see Supplementary Information).

**Fig 8:**
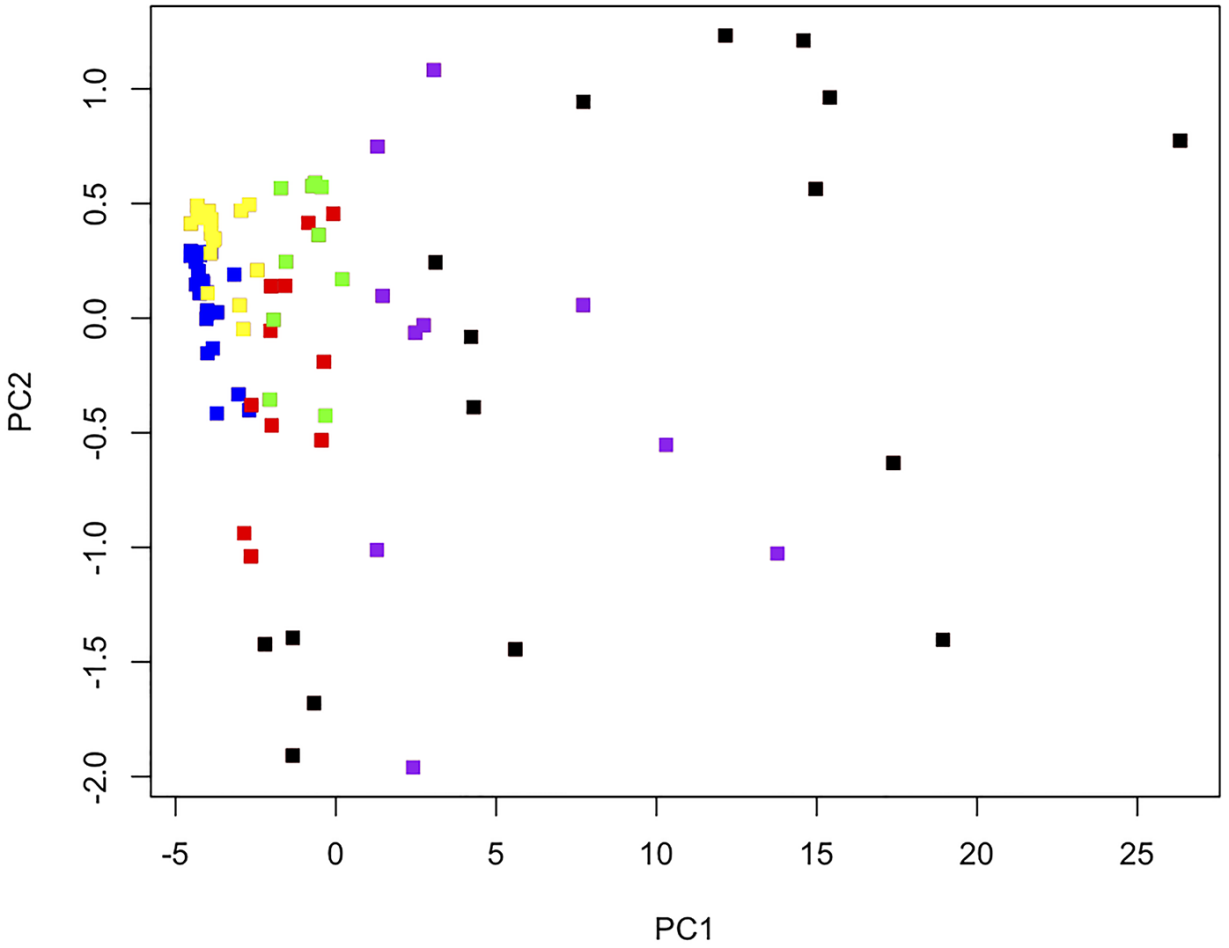
PCA plot of principal components 1 and 2. PCA plot of principal components 1 and 2, colour-coded by observation category, i.e. control versus IL-1 stimulated and range of initial cytoplasmic fluorescence. The six categories are: IL-1 stimulated/0-1.5 = Blue, IL-1 stimulated/1.5-3.0 = Red, IL-1 stimulated/>3.0 = Black, control/0-1.5 = Yellow, control/1.5-3.0 = Green, and control/>3.0 = Purple. The plot shows separation of observations with initial fluorescence < 1.5 units from the rest of the data, with partial separation between the control and IL-1 stimulated observations within this group. There is also a limited degree of separation between observations with initial fluorescence values of 1.5-3.0 units from the rest of the data, however the amount of overlap between control and IL-1 stimulated is more significant here.

Subsequent analysis was performed on the four principal components, with the individual observations being coded depending on the relevant category. Initial comparisons representing *control* and *IL-1 stimulated* observations did not yield separation of observations. Additional granularity of coding the individual observations allowed us to compare stimulation status (control and IL-1 stimulated) against ranges of the cytoplasmic fluorescence data expressed relative to levels at time_0_. The best separation occurred using initial fluorescence ranges of 0-1.5 fluorescence units, consistent with the biological analysis by Carlotti et al [29] and Yang et al [31, 32]. Complete separation does not occur for any combinations, however separation emerges between control and stimulated conditions for cells with initial cytoplasmic fluorescence up to 1.5 fluorescence units. Fig 8 represents the plot of PC1 versus PC2, which separates control and IL-1 stimulated observations grouped by their initial cytoplasmic fluorescence. There is limited separation between control and IL-1 stimulated cells with initial fluorescence levels of 1.5-3.0 and no appreciable difference at initial fluorescence > 3.0 units using PCA. This accurately reproduces the findings of Yang et al [31, 32], which showed reduced activity at concentrations above 1.5 fluorescent units. Similarly, Carlotti et al [29] demonstrated that lag time and nuclear translocation rate of the transcription factor are markedly decreased at higher concentrations and that, the subsequent step involving nuclear translocation of NF-*κ*B, was completely blocked at fluorescence units > 3.0. This likely reflects the well-controlled system of feedback mechanisms regulating the NF-*κ*B signalling pathway.

### Modelling Component-Level Interactions

This medium-level abstraction decomposes the IL-1 stimulated NF-*κ*B signalling pathway into its constituent molecular components. This level models an abstracted view of the various molecular interactions between the components, which together give rise to the emergent behaviours of the system.

#### Modelling the Cascade of Interactions

**Fig 9:**
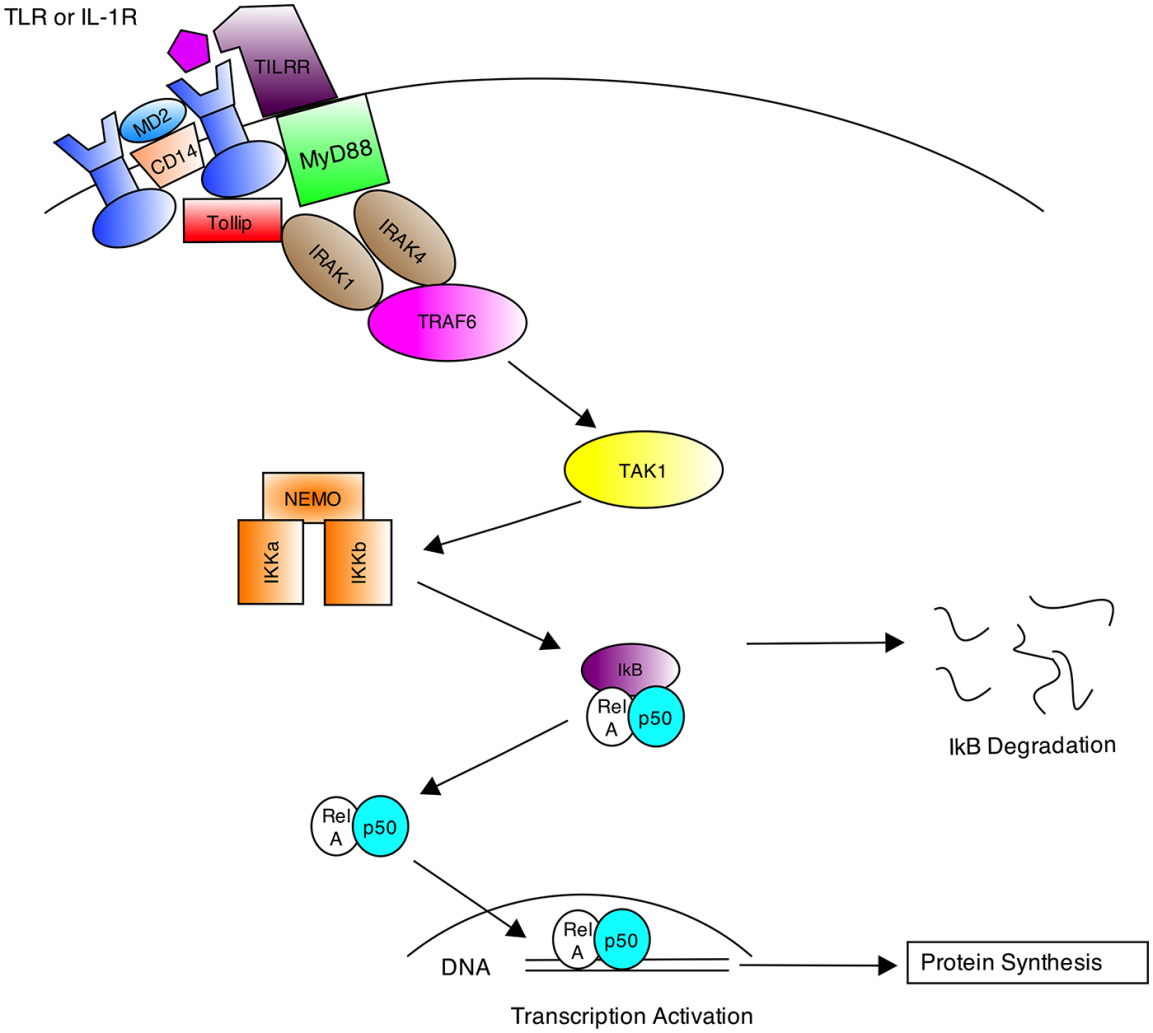
Cartoon diagram of high-level interactions. Simplified cartoon diagram depicting the high-level interactions between the TLR or IL-1R superfamily of receptors, the co-receptors and adaptor proteins, and the protein kinases within the NF-*κ*B canonical signalling pathway. Diagram developed from findings of [51–55].

**Fig 10:**
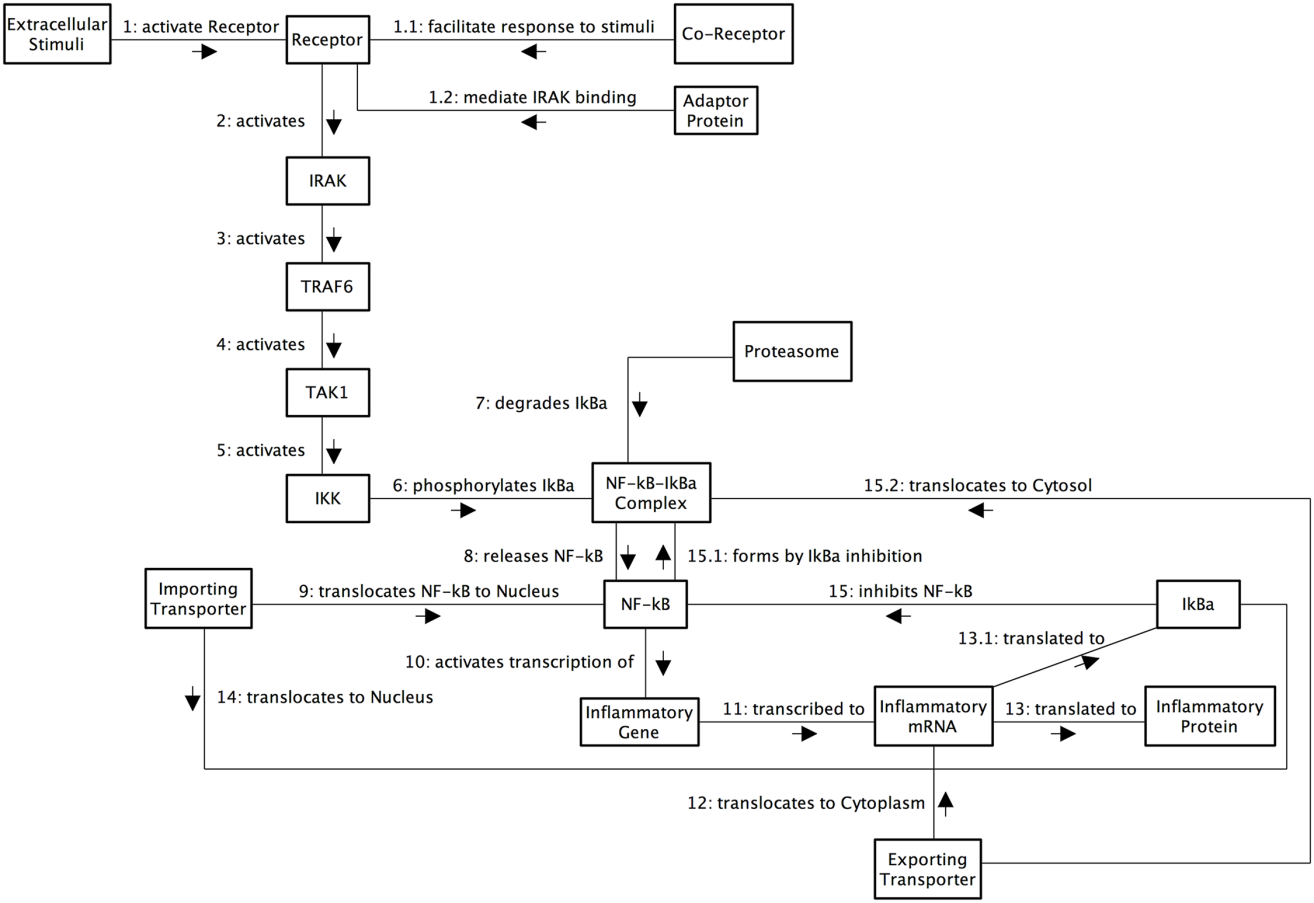
UML communication diagram. UML communication diagram for the IL-1 stimulated NF-*κ*B signalling pathway. Although portraying temporal interactions as per sequence diagrams (not shown), we believe that these diagrams are more intuitive for non-Computer Science audiences as they are more flexible in relation to the position of system components, thus allowing the positioning of components to approximate to the spatial locations within a Eukaryotic cell. Developed from reviews of [56, 57].

As per the system-level properties, the NF-*κ*B signalling pathway can be described from a high-level perspective using cartoon diagrams to communicate the interactions between system components, and in this instance the diagram can also act as a network map and illustrate the sequence of interactions between components (see Fig 9). The UML *communication* diagram (see Fig 10) builds on this high-level cartoon to convey the network map in a more formalised way.

Briefly, the network commences with an extracellular ligand (signalling molecule) binding to a cell membrane receptor (which is a member of the TLR/IL-1 receptor superfamily). The receptor then dimerises, and co-receptors such as CD14 [58], MD2 [59] (in the case of TLR4, [60]) and TILRR [55, 61] (in the case of IL-1RI/IL-1AcP) help facilitate and amplify the receptor response. In situations where the Tollip adaptor protein binds, it mediates association of IRAK protein kinase to the IL-1 receptor complex, but then inhibits IRAK [52] and transduction of the signal down the signalling pathway. Conversely, in situations where the MyD88 adaptor protein binds, it mediates association of the receptor complex with IRAK protein kinase [62], which in turn activates TRAF6 through phosphorylation [63] for propagation of the signal. Once activated, TRAF6 continues signal transduction through activation of TAK1, which subsequently activates the IKK complex [53, 54]. The activated IKK complex phosphorylates NF-*κ*B inhibitors, such as I*κ*B*α*, which facilitates its dissociation from the NF-*κ*B molecule within the complex [64]. The released I*κ*B*α* undergoes a second modification called polyubiquitination [65], which then targets I*κ*B*α* for rapid degradation by the proteasome. Conversely, the released NF-*κ*B is able to translocate from the cytosol to the nucleus, where it is subsequently activated and upregulates the transcription of target genes.

#### Modelling Activities

**Fig 11:**
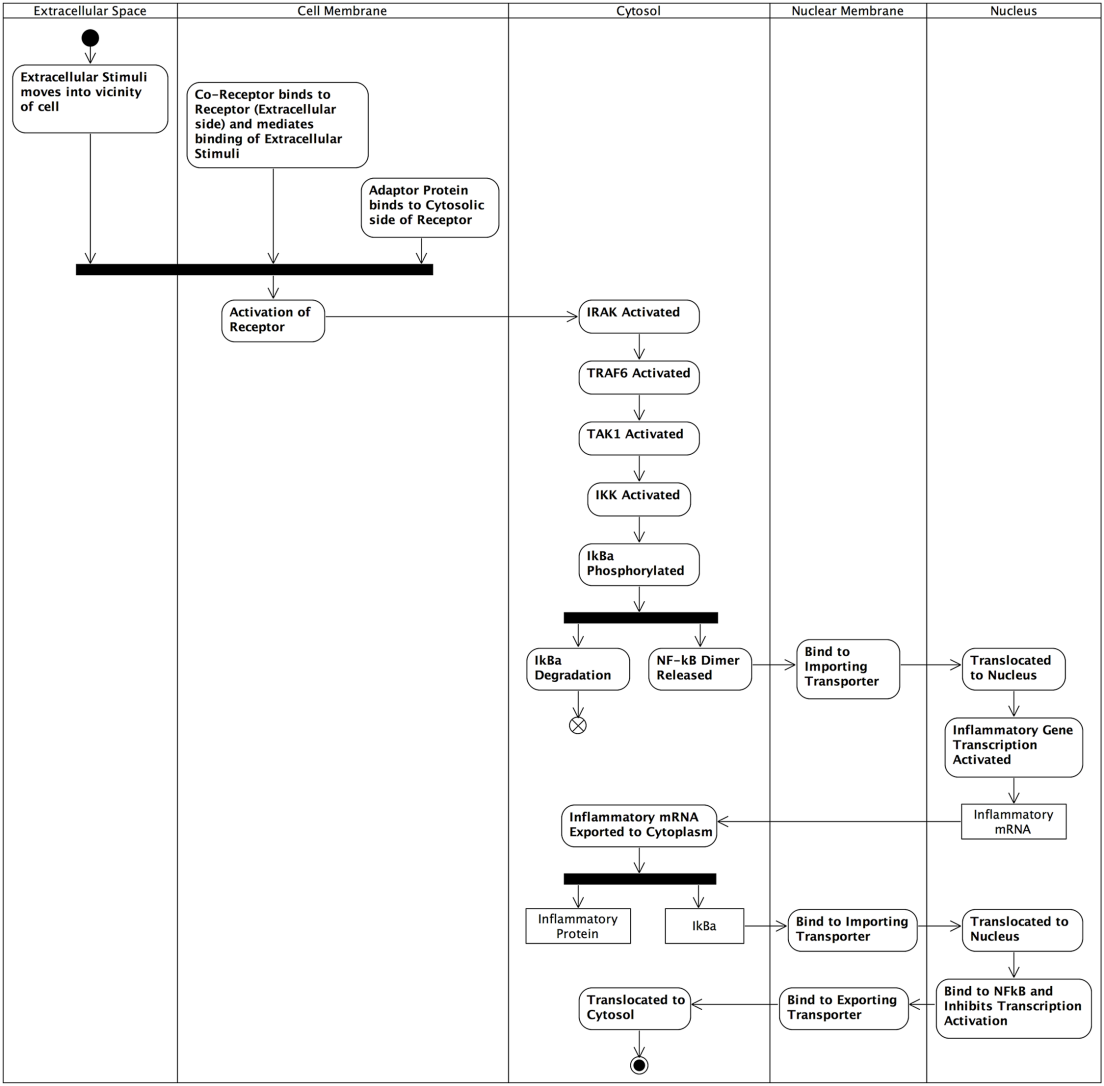
UML activity diagram. Full end-to-end UML activity diagram for the IL-1 stimulated NF-*κ*B signalling pathway using the concept of swim-lanes to convey sub-cellular location of components. Developed from [39, 56, 57].

Another complementary UML notation that provides a view of activities within the system instead of component interactions is the *activity* diagram, which through the use of *swim-lanes* may also be used to convey the location of activities (see Fig 11). As activity diagrams focus on activities and not components, they are able to convey the individual interactions (expressed as activities) that give rise to the emergent behaviour of the system. Furthermore, the focus on activities allows us to aggregate sets of individual interactions into functional modules. This is beneficial when domain modelling, as biological systems can generally be abstracted into groupings of components by functionality. With particular reference to the IL-1 stimulated NF-*κ*B signalling pathway, we can separate the system into three functional modules relating to cell membrane receptor activation, activation of the NF-*κ*B signalling module, and generation of new I*κ*B*α* to dampen the response through negative feedback regulation.

As per the cartoon network diagram (Fig 9) and UML communication diagram (Fig 10), the set of activities within the system begin with extracellular stimuli and the formation of the active receptor complex. The associated signal transduction then follows, with the first activity being the activation of IRAK, which then propagates the stimuli-related signal through the pathway via phosphorylation of intermediates. Upon phosphorylation of the I*κ*B*α* inhibitor which is bound to NF-*κ*B, the activity splits into two branches: a) phosphorylated I*κ*B*α* is released from the NF-*κ*B complex and becomes degraded via the proteasome, and b) the NF-*κ*B dimer is released, binds to an importing nuclear receptor, is translocated from the cytosol to the nucleus, and is then activated. Once active, the NF-*κ*B dimer may bind to the promoter region of an inflammatory response gene and initiate transcription, which ultimately generates new inflammatory response proteins.

### Modelling Individual Component Dynamics

**Fig 12:**
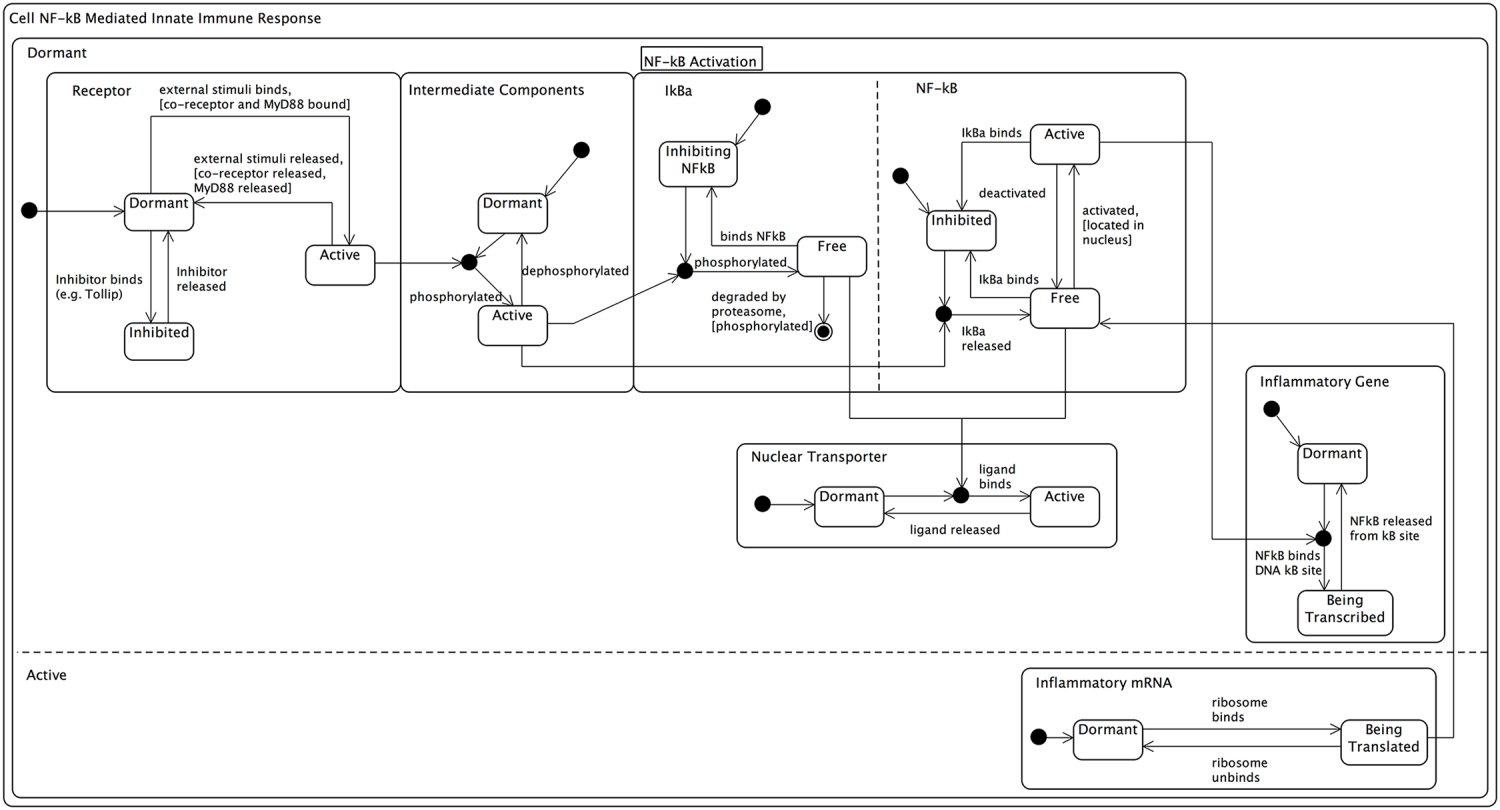
Linked state machine diagrams. Linked series of state machine diagrams for the IL-1 stimulated NF-*κ*B signalling pathway. The individual components have their own state machines, which are explicitly linked using UML join notations and embedded within a single large state machine that represents the cell. Here the cell has two states relating to dormant or active. Developed using [41, 52, 56, 57, 66–68].

This level of abstraction provides the greatest detail within the domain model, through modelling the dynamics of individual components within the system. The final set of UML diagrams that represent the domain model, refer to low-level dynamics of individual components and use the *state machine* diagram notation. Fig 12 depicts a set of linked state machine diagrams for the receptor, intermediate components, I*κ*B*α*, NF-*κ*B, nuclear transporter, inflammatory gene, and inflammatory mRNA components of the signalling pathway. We believe the ability to link individual state machine diagrams into a single end-to-end diagram provides a powerful approach for domain modelling, as it allows the low-level dynamics of components to be captured in a single diagrammatic view of the system as a whole.

It can be seen that the cell membrane receptor initially starts off in a *dormant* state, but may become *active* upon binding of extracellular stimuli, along with the co-receptor and MyD88 adaptor protein (defined using UML guard notation). Conversely, and as discussed previously, the cell membrane receptor may also become *inhibited* upon binding of Tollip. As defined in the previous cartoon and UML diagrams, following activation of cell membrane receptor, the extracellular signal is propagated through the signalling pathway through activation of intermediate components, culminating with activation of IKK. For the purposes of the domain model, we have abstracted away the granularity of these intermediate components (e.g. IRAK, TRAF6, TAK1 and IKK) to that of a *generic* intermediate component, which by default is *dormant*, but becomes *active* following phosphorylation as the signal is propagated through the transduction cascade.

Within the NF-*κ*B signalling module, the I*κ*B*α* inhibitor molecule by default (i.e. following creation via transcription and translation) is unbound (*free*), but may probabilistically bind to NF-*κ*B when it enters an interaction boundary, and therefore enters an *inhibiting* state. Following activation of the IKK enzyme, the I*κ*B*α* releases the NF-*κ*B dimer, to again enter the *free* state, but this time is degraded and removed from the system. Similarly, the NF-*κ*B dimer, is by default in an *inhibited* state within the system due to I*κ*B*α* inhibition. Following IKK-mediated release by I*κ*B*α*, it becomes *free*, and able to translocate to the nucleus where it may become *active* (note the guard condition), to facilitate the upregulation of inflammatory gene transcription. Should the NF-*κ*B dimer spontaneously unbind from the promoter region of the inflammatory gene, it will once again enter the *free* state (upon which it may probabilistically translocate back to the cytoplasm), or alternatively new I*κ*B*α* molecules, this time within the nucleus may also bind to return the NF-*κ*B dimer to an *inhibited* state, upon which the nuclear localisation sequence will be masked and it will be translocated out of the nucleus into the cytoplasm [39].

As per previous UML diagrams, binding of an I*κ*B*α* molecule or NF-*κ*B dimer to a nuclear membrane transporter, transitions the transporter protein from a *dormant* to an *active* state, for the translocation of the ligand from either the cytoplasm to the nucleus, or vice versa. Following translocation of an NF-*κ*B dimer to the nucleus and its binding to the promoter region of an inflammatory gene, the gene transitions from a *dormant* to an actively *being transcribed* state for generation of mRNA. Upon creation, the new mRNA is translocated to the cytoplasm, where it is *translated* into new inflammatory protein by the ribosome.

### Modelling Numerical Aspects of the System

The three different views outlined above provide a top-down perspective of the IL-1 stimulated NF-*κ*B signalling pathway, which reflects the hierarchical nature of complex systems. The UML and cartoon-like diagrams used so far, have been useful for semi-formally defining the relationships and dynamics at the system, component, and intra-component levels, however they have not been able to appropriately capture the numerical aspects of the signalling pathway. For example, we have found diagrammatic notations to be deficient in modelling details regarding the ratios of NF-*κ*B molecules (in *free* and *inhibited* states) against *free* I*κ*B*α* molecules across the cytoplasmic and nuclear compartments. Similarly, we have been unable to convey nuclear translocation dynamics or details of I*κ*B*α* degradation within UML in a form that would be intuitive to biologists. Table 1 therefore defines the key rates, ratios, and physical attributes associated with the IL-1 stimulated NF-*κ*B signalling pathway.

**Table 1:** The key rates, ratios and constants.

High-Level Attribute	Specific Attribute	Value
Cell Environment	Cell Volume	2,000 *μ*m^3^
	Nucleus Volume	100 *μ*m^3^
Approx. No. per Cell	IL-1RI Receptors	5,000—10,000
	RelA (NF-*κ*B)	60,000 (Endogenous)
	I*κ*B*α*	66,000 (Endogenous), ∼135,000 (Endogenous, cytoskeleton-bound)
NF-*κ*B	Cytoplasmic:Nuclear Location	10:1
	Bindable NF-*κ*B: I*κ*B*α*	1:1 ratio; ∼17% NF-*κ*B ‘free’ in resting cells
	Total NF-*κ*B: I*κ*B*α*	1:3 (including cytoskeleton sequestered)
	IL-1 Stimulated	∼20% decrease in cytoplasmic NF-*κ*B; ∼40-fold increase in nuclear NF-*κ*B; ∼8-fold increase in transfected v endogenous NF-*κ*B
I*κ*B*α*	Phosphorylation	Peaks at 10 min post IL-1 stimulation
	Ubiquitination	Peaks at 30 min post IL-1 stimulation
	Degradation	∼40% degraded after 10 min; ∼60% degraded after 30 min; ∼80% degraded after 60 min
Nuclear Translocation	NF-*κ*B flow to nucleus	40-60 molecules/sec (max IL-1 stimulation); Following nuclear NF-*κ*B peak, takes ∼90 min to reach basal steady-state cytoplasm: nucleus ratio
	Shuttling	Dissociation of NF-*κ*B-I*κ*B*α* complex within cytoplasm and independent import of subunits; Continuous process, i.e. steady-state dynamics of in- and out- fluxes; Lag time in nuclear translocation following IL-1 stimulation; Negligible NF-*κ*B-I*κ*B*α* complex translocation; I*κ*B*α* translocation more rapid than NF-*κ*B; After IL-1 stimulation, increased rate of nuclear to cytoplasmic translocation

The key rates, ratios and constants of the IL-1 stimulated NF-*κ*B signalling pathway. This table provides key details for the cell environment, approximate number of key molecules, I*κ*B*α* biochemistry, nuclear translocation, and ratios of NF-*κ*B and I*κ*B*α* molecular states within the cytoplasm and nucleus. Developed from the work of [27, 29–32].

## Discussion

Biological systems are complex, with behaviours and characteristics that result from a highly connected set of interaction networks that function through time and space. As discussed previously, the IL-1 stimulated NF-*κ*B signalling pathway is a complex intracellular network that manifests in stochastic and dynamic responses to inflammatory stimuli. The system-wide behaviours, generated as an inflammatory response to pathogenic invasion and other physiological perturbations, emerge through the cumulative effect of low-level intracellular interactions within an individual cell, being amplified across a population of immune response cells. As such, the inherent complexity of the signalling pathway and its associated stochasticity and dynamics, renders the process of domain modelling both time consuming and non-trivial in nature.

Being analogous to a functional specification (from software engineering), the primary purpose of the domain model is to clearly and unambiguously capture our abstracted view of the functionality of the real-world domain, which will be incorporated within the future iterations of the resulting computational model. We found the iterative process of domain modelling to be extremely helpful in allowing the modeller to explore the biological domain (in conjunction with the *domain expert*) before development of the computational model. Once complete, and validated, the domain model acts as the functional specification for the computational model, and provides a comprehensive and transparent understanding of the domain that underpins both the scope of the computational model and the resulting *in silico* experimentation that will be performed as part of the exploration phase of the CoSMoS project. As such, the domain model is an essential project deliverable that provides an audit trail on how the real-world biology is linked, through abstractions, assumptions, and constraints to the functionality of the computational model. Furthermore, in this specific case, the actual process of developing the domain model in conjunction with the domain expert, facilitated a much more in-depth understanding of the domain than would have been gained through published literature alone.

The domain model may be a collection of informal notes relating to relevant aspects of the domain, but may also include informal sketches (such as cartoons), more formal diagrams (such as those produced with UML), mathematical equations, scientific constants (e.g. biochemical rate constants), and physical descriptors (such as size, quantity, location, and speed). The key constraint of the domain model is that it should remain free from an implementation specific focus and should therefore not contain any reference to the programming languages or workarounds, which may be required during development of the simulator. As such, the domain model should be focused on the scientific domain, and not design considerations for the resulting computational model. Through this approach of using cartoon diagrams, UML notation, statistical techniques, and descriptions of rates, ratios and constants within a table, we have developed an accurate reproduction of the biological domain.

There exists substantial quantities of literature on NF-*κ*B signalling dynamics, and various aspects of the signalling dynamics are independently studied by a wide variety of labs. It is generally understood that representing every aspect of a real-world system in models and simulations is computationally intractable. As such, a subset of the properties and behaviours from the real-world system need to be defined for subsequent investigation. One of the primary purposes of the domain model is to capture this subset of real-world system properties, at the correct abstraction level to answer the questions of scientific interest; for example, the single-cell data relating to NF-*κ*B pathway dynamics, mean that our future computational model should be at the level of subcellular interactions and biochemical reactions within a single cell.

Due to the complex, stochastic nature of the IL-1 stimulated NF-*κ*B signalling pathway, we have been unable to develop a single diagrammatic view that could capture the various components, interactions, and dynamics of the system. It has therefore been necessary to utilise a number of different cartoons and UML notations throughout our domain modelling exercise. These different diagrammatic views allow us to capture the initiation and propagation of the signalling pathway across the inherent hierarchies of the system (i.e. system-wide, component interactions, and individual component dynamics), which we believe to be a natural progression when domain modelling and reflects the concepts of hierarchy and modularity from systems biology [69]. The use of cartoon and UML diagrams were an essential first step towards development of our domain model, however in isolation they were not enough to provide a comprehensive model. In particular, they were unable to convey the dynamics of I*κ*B*α* degradation (along with the associated NF-*κ*B release and subsequent activation), or indeed model the quantitative aspects of the signalling pathway. We therefore used a number of descriptive and multivariate statistical techniques to complement the UML diagrams, in order to develop a more comprehensive domain model of the IL-1 stimulated NF-*κ*B signalling pathway. Through this use of statistical techniques to complement UML, we have successfully reproduced the system interactions and stochastic dynamics found in biology.

Two tailed *χ*^2^ goodness of fit tests were used due to the uncertainty about direction of difference of the observed versus expected data. The full dataset has been shown to approximate to a Negative Binomial distribution (see Figs 5 and 6), which is in keeping with the findings of White and Bennetts [70] and Bliss and Fisher [46] who advise (through statistical modelling of a number of biological systems) that biological populations, be that cell or organism level, often approximate very closely to negative binomial distributions. There is also a subset of observations within the 0-3.0 fluorescence units range however, which tend to Normality when using non-integer binning frequencies (not shown). As such, future statistical tests on the data, and indeed any simulation-level data produced from *in silico* experimentation, should be non-parametric in nature as these are applicable to any distribution, and do not assume normality. Furthermore, due to its non-parametric nature, the central measure used should be the median average, as this is not affected to the same extent from skewed data as the mean average [71].

Additionally, we have shown that multivariate techniques may be used to classify single-cell analysis observations into groups dependent on their initial fluorescence, and to separate control from IL-1 stimulated observations within ranges of fluorescence units. Through hierarchical cluster analysis, and analysing the various PCA plots (for example, the PC1 v PC2 plot in Fig 8), it can be deduced that there is evidence of partial separation of control versus IL-1 stimulated observations, beyond which the inherent variance associated with the data becomes too great. We accept that the large degree of variation is consistent with normal biology, however believe that for the purposes of our research, we should focus on a subset of experimental data. As the advantage of single-cell analysis is lost if you pool the data and calculate an average, and with the results of the above multivariate statistical tests in mind, we propose that a series of expression levels are used for development and calibration of our future computational model. This approach agrees with earlier findings by Carlotti et al [29] who advised that cells with high expression of the enhanced green fluoresecent protein and NF-*κ*B (RELA) construct show impaired nuclear translocation dynamics, and that these aberrant cells mask the dynamics (at the population level) of cells expressing near-physiological amounts of the fusion protein. We also believe that in order to get rational results, each cell needs to form its own control (which was also the approach taken for the model of Pogson et al [28]), in order to eliminate the wide variations observed when averaging dynamics over multiple cells, and by implication simulations. Furthermore, it is believed that such an approach would yield more consistent results as cell time-course dynamics would be expressed as a percentage of initial fluorescence for each cell.

As per Read et al [72] and Bersini [73], we agree that a subset of UML notations are able to efficiently represent elements of the domain model of biological systems (in our case the IL-1 stimulated NF-*κ*B signalling pathway). We have found activity diagrams and communication diagrams particularly effective at depicting system-wide behaviours; communication diagrams to be effective at depicting relationships between components; and state machine diagrams effective at depicting low-level dynamics within individual components. Furthermore, we have found that activity diagrams are particularly effective when used in conjunction with swim-lanes to convey the location (e.g. cytoplasm or nucleus) of activities, and sequentially linked state machine diagrams are particularly effective at depicting the end-to-end state changes within a system.

Although we have found UML to be particularly useful in these cases, it does have a number of deficiencies however. Along with the issues found by Read et al [72], we have discovered a number of additional areas where the current UML standards have deficiencies in modelling biology. For example, although UML facilitates detailed information to be depicted as attributes of individual components, it relies on the reader to unpick the multitude of diagrams to collate all of the information, for example parameter values (such as size of cell, and speed of movement of intracellular components) and rate constants (such as degradation of I*κ*B*α*, and translocation of components across the nuclear membrane). We believe that a table of such information would provide a more effective mechanism to convey this information, than to over-engineer a UML diagram. Furthermore, although UML allows the range of individual objects to be depicted through multiplicity, in the form of a zero to many ‘0‥*’ association, this does not effectively convey the degree of simultaneous interactions between agents. Similarly, it is well understood that observations of genetically identical, individual cells in a standardised environment often display significant differences in their response to perturbations [44], thus leading to the large degree of *inherent* variation within biological populations (be they cells, organisms, or communities). At a molecular level, this may be due to the varying numbers of particular proteins within a population of cells. UML does not have the ability to depict this variation, and nor was it designed to.

## Conclusions

In this article, we have presented a domain model of the IL-1 stimulated NF-*κ*B signalling pathway using UML and statistical techniques. UML has been advocated as a modelling language for visualization, specification, construction and documentation of software systems [74]. Although it is not a programming language, we believe that together with a modelling approach (such as agent-based modelling) and programming language (such as Java or C), UML provides an excellent mechanism to develop models of complex dynamical systems. We agree with Cook [75] that “UML is likely to influence model-driven development for the foreseeable future”, but through adoption of a principled approach to development of our domain model, we have discovered that UML has a number of deficiencies when trying to convey the stochastic, heterogeneous nature of dynamics, within complex biological systems. This lack of functionality leads us to conclude that UML should not be seen as the only tool to be used in the domain modelling process. We address this problem by utilising a number of statistical techniques in order to gain a fuller understanding of the domain, and for scoping the abstraction of the domain to be taken forward into our future computational model. Likewise, UML does not currently have the ability to depict patterns within wet-lab data, which we believe is an essential component of the domain model for complex biological systems.

It is generally agreed [76–78], that the principled design and development of a conceptual model (such as the Domain Model in the CoSMoS approach) is an essential step towards ensuring the *right* computational model is developed. As such, our domain model represented here will serve as evidence during validation and verification of the resultant computational model (Simulation Platform) that will be developed during the exploration phase of our CoSMoS project (forthcoming). Our multi-level domain model has taken a top-down approach by looking at the emergent system-wide behaviour, followed by component interactions, and finally the individual component dynamics. As such, within our domain of interest, the statistical techniques have been used at the system-level only, due to the wet-lab (*in vitro*) data being based on fluorescence at the single-cell level; the other levels within our multi-level domain model have therefore been developed using UML diagrammatic notations. We therefore believe that multi-level domain models developed using UML, benefit from the complementary views that emerge from statistical analysis of the underlying *in vitro* data. We acknowledge however, that the ability of statistical techniques to model any interplay between the different levels within a multi-level system, is reliant on characteristics of the empirical (i.e. wet-lab) data.

Rumpe and France [79] advise that different stakeholders and modellers from different domains have varying interpretations of what constitutes an appropriate UML diagram. They further advise that as the UML specification allows the modeller a degree of flexibility through the use of semantic variations, diagrams can be tailored to better support the varied requirements of individual modellers, stakeholders, and their respective domains. We therefore suggest that the statistical techniques used within this case study, along with the various cartoon-like diagrams for modelling the expected behaviours of the system (see Fig 3) and physical containment of components (see Fig 4) represent an example semantic variation point for modelling complex intracellular signalling pathways.

Finally, we believe that community and industry standards, such as UML, are important for improving the communication between developers and domain experts. The use of these standards, should make the reimplementation of models by different researchers (and labs) easier, and indeed should reduce the duplication of work, and more importantly reduce implementation errors, which may become introduced through reverse engineering of existing models and manual walkthroughs of published papers. We therefore believe the use of cartoon and UML diagrams to be an essential first step towards development of a domain model, which may be published alongside the results of *in silico* experimental papers; however in isolation they are not enough to provide a comprehensive model, which other researchers and labs may use to reproduce computational models. In particular, cartoons and UML diagrams have been unable to convey the dynamics of I*κ*B*α* degradation (along with the associated NF-*κ*B release and subsequent activation), or indeed model the quantitative aspects of the signalling pathway. We therefore conclude that the use of descriptive and multivariate statistical techniques to complement the UML diagrams, is essential for the development of comprehensive domain models of complex biological systems, such as the IL-1 stimulated NF-*κ*B signalling pathway. Indeed, through our principled approach for domain modelling, we have accurately reproduced the stochastic nature of the real-world system using a diagrammatic and statistical approach.

## Supporting Information

S1 Fig Dendrogram representing the clustering of single-cell analysis observations. Dendrogram representing the clustering of observations from [31] by hierarchical cluster analysis using the complete(-linkage) method. The boxes indicate that hierarchical cluster analysis identifies the three forced clusters as observations having an initial cytoplasmic fluorescence less than 3.0, between 3.0 and 8.0, and above 8.0 fluorescence units.
(TIF)

S2 Fig Scree plot of the principal components from principal component analysis of the single-cell fluorescence data. Scree plot of the principal components from principal component analysis of observations from Yang et al [32]. Each bar corresponds to its respective principal component; bar heights are the variances of the principal components.
(TIF)

S3 Fig Bi-plot of PC1 and PC2 from principal component analysis of the single-cell fluorescence data. Bi-plot of PC1 and PC2 from principal component analysis of observations from Yang et al [32]. This plot shows that measurements for times 0, 10 and 30 min contribute equally to the separation of PC1 due to their virtually equivalent arrow lengths. They are not fully parallel to the PC1 axis however, and therefore also contribute slightly to PC2.
(TIF)

S4 Fig Plot of loadings for PC1 following principal component analysis. Plot of loadings for principal component 1 following PCA. PC1 was chosen because this is the component which contributes most to separation of the data. It can be seen that observations with initial fluorescence between 0-3.0 and >3.0 can be separated easily as the observation between 0-3.0 units have negative loadings and >3.0 have positive loadings. Furthermore, observations for cells with initial fluorescence between 0-1.5 tend to have relatively stable loadings (around -4.5), whereas those between 1.5-3.0 begin to have more variable loadings.
(TIF)

S1 Table Control observations for data analysis. Subset of control observations from the single-cell analysis of Yang et al [32] that were used within our data analysis.
(PDF)

S2 Table IL-1 stimulated observations for data analysis. Subset of IL-1 stimulated observations from the single-cell analysis of Yang et al [32] that were used within our data analysis.
(PDF)

S3 Table *χ*^2^ test for control observations. *χ*^2^ test for control observations approximating to a negative binomial distribution.
(PDF)

S4 Table *χ*^2^ test for IL-1 stimulated observations. *χ*^2^ test for IL-1 stimulated observations approximating to a negative binomial distribution.
(PDF)

S5 Table Summary of principal component analysis of the single-cell fluorescence data. Summary of principal component analysis of the single-cell fluorescence data, showing the standard deviation, proportion of variance and cumulative proportion of variance for each principal component.
(PDF)

S1 File Subset of Single-Cell Observations used within our Domain Model. (PDF)

S2 File Chi-squared (*χ*^2^) goodness of fit. (PDF)

S3 File Hierarchical Cluster Analysis. (PDF)

S4 File Principal Component Analysis. (PDF)
