# Supplementary figures and images for: Statistical Techniques Complement UML When Developing Domain Models of Complex Dynamical Biosystems
Source: PLoS One. 2016 Aug 29;11(8):e0160834. doi: 10.1371/journal.pone.0160834 (PMC5003378; doi:10.1371/journal.pone.0160834)

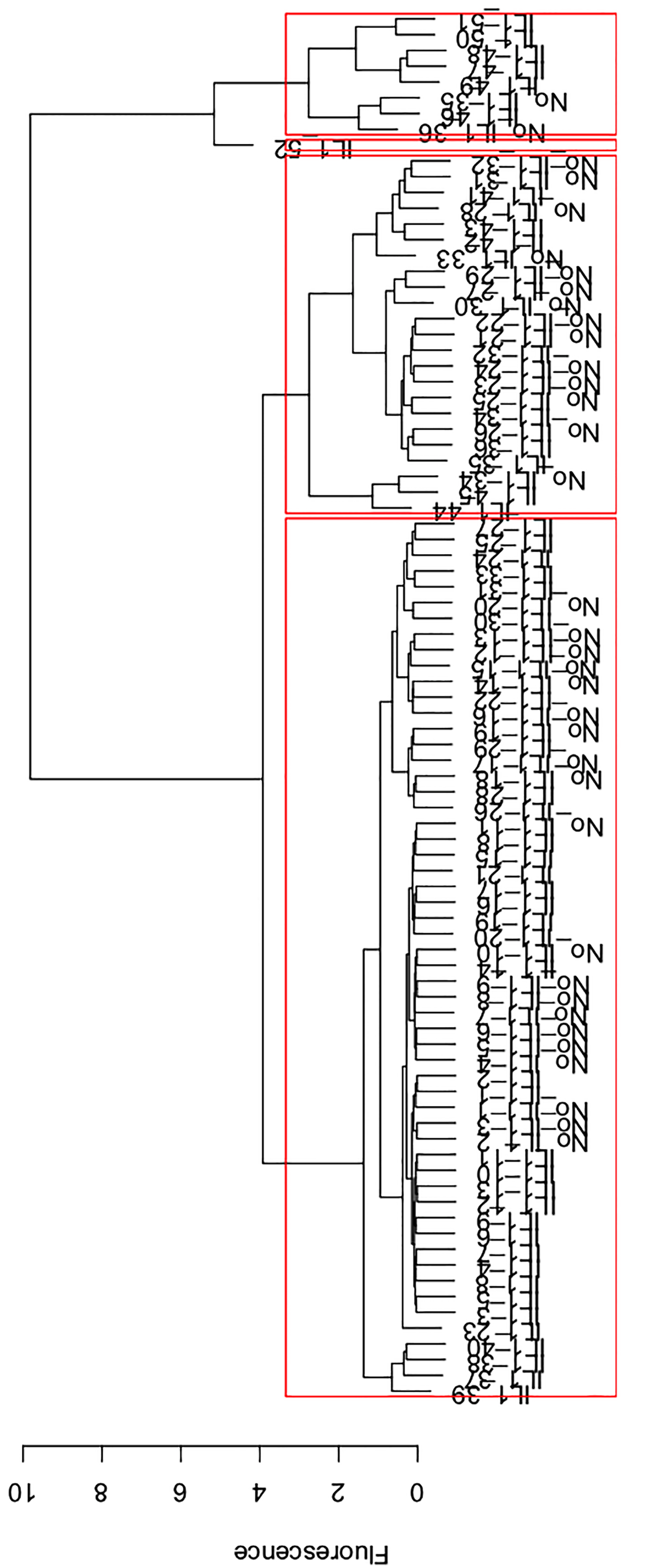

Supplement: S1 Fig — Dendrogram representing the clustering of observations from [31] by hierarchical cluster analysis using the complete(-linkage) method. The boxes indicate that hierarchical cluster analysis identifies the three forced clusters as observations having an initial cytoplasmic fluorescence less than 3.0, between 3.0 and 8.0, and above 8.0 fluorescence units. (TIF) [file pone.0160834.s001.tif]

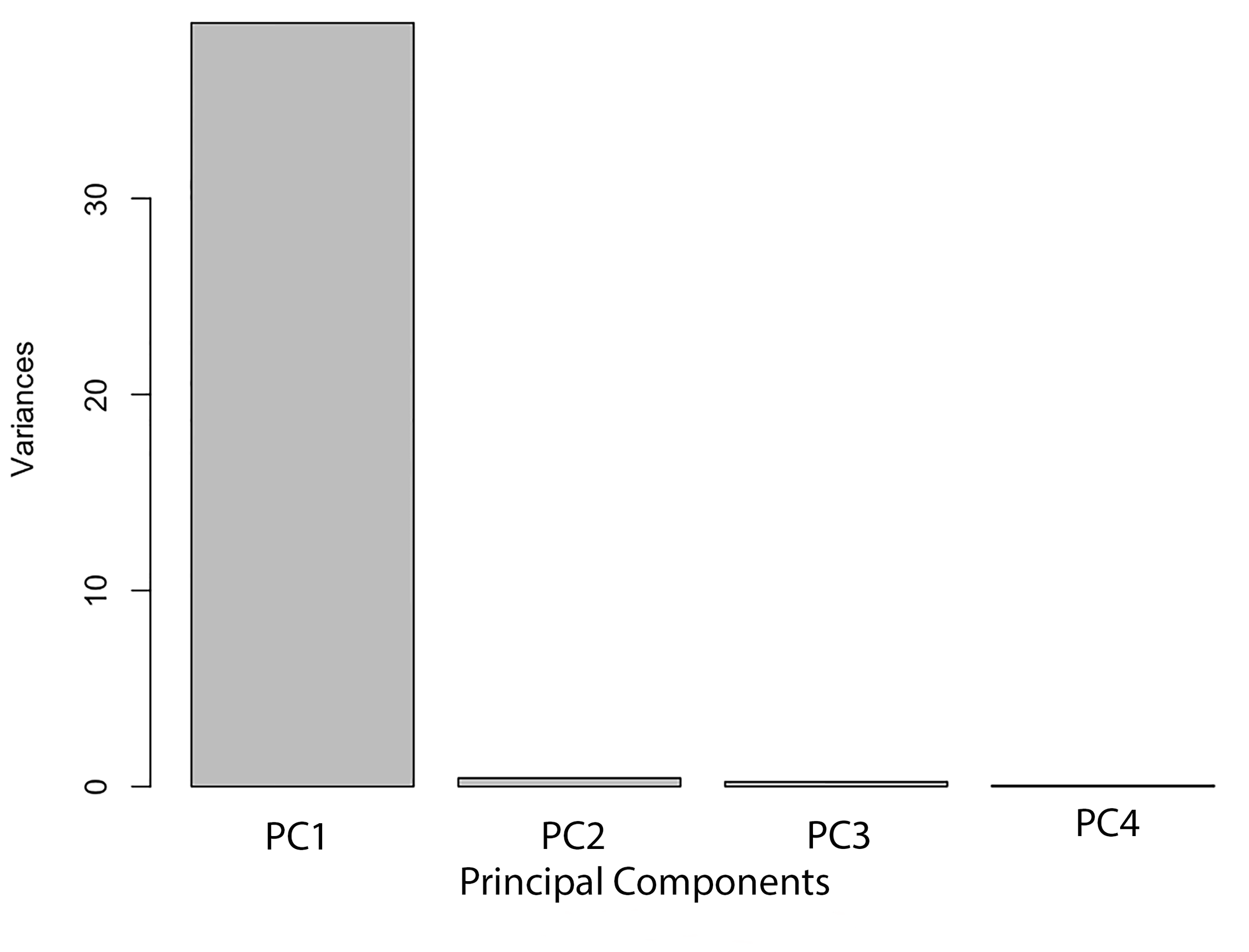

Supplement: S2 Fig — Scree plot of the principal components from principal component analysis of observations from Yang et al [32]. Each bar corresponds to its respective principal component; bar heights are the variances of the principal components. (TIF) [file pone.0160834.s002.tif]

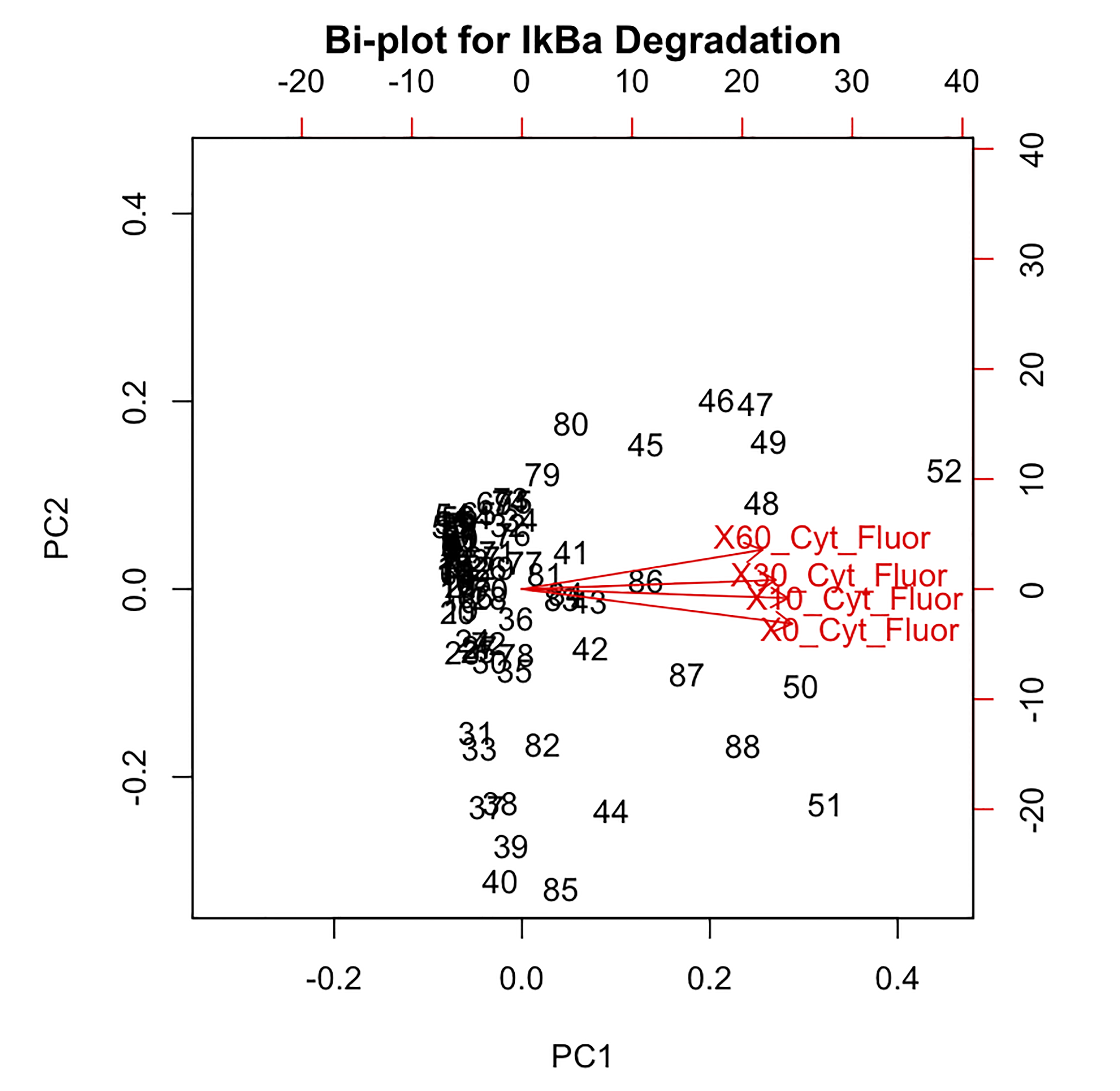

Supplement: S3 Fig — Bi-plot of PC1 and PC2 from principal component analysis of observations from Yang et al [32]. This plot shows that measurements for times 0, 10 and 30 min contribute equally to the separation of PC1 due to their virtually equivalent arrow lengths. They are not fully parallel to the PC1 axis however, and therefore also contribute slightly to PC2. (TIF) [file pone.0160834.s003.tif]

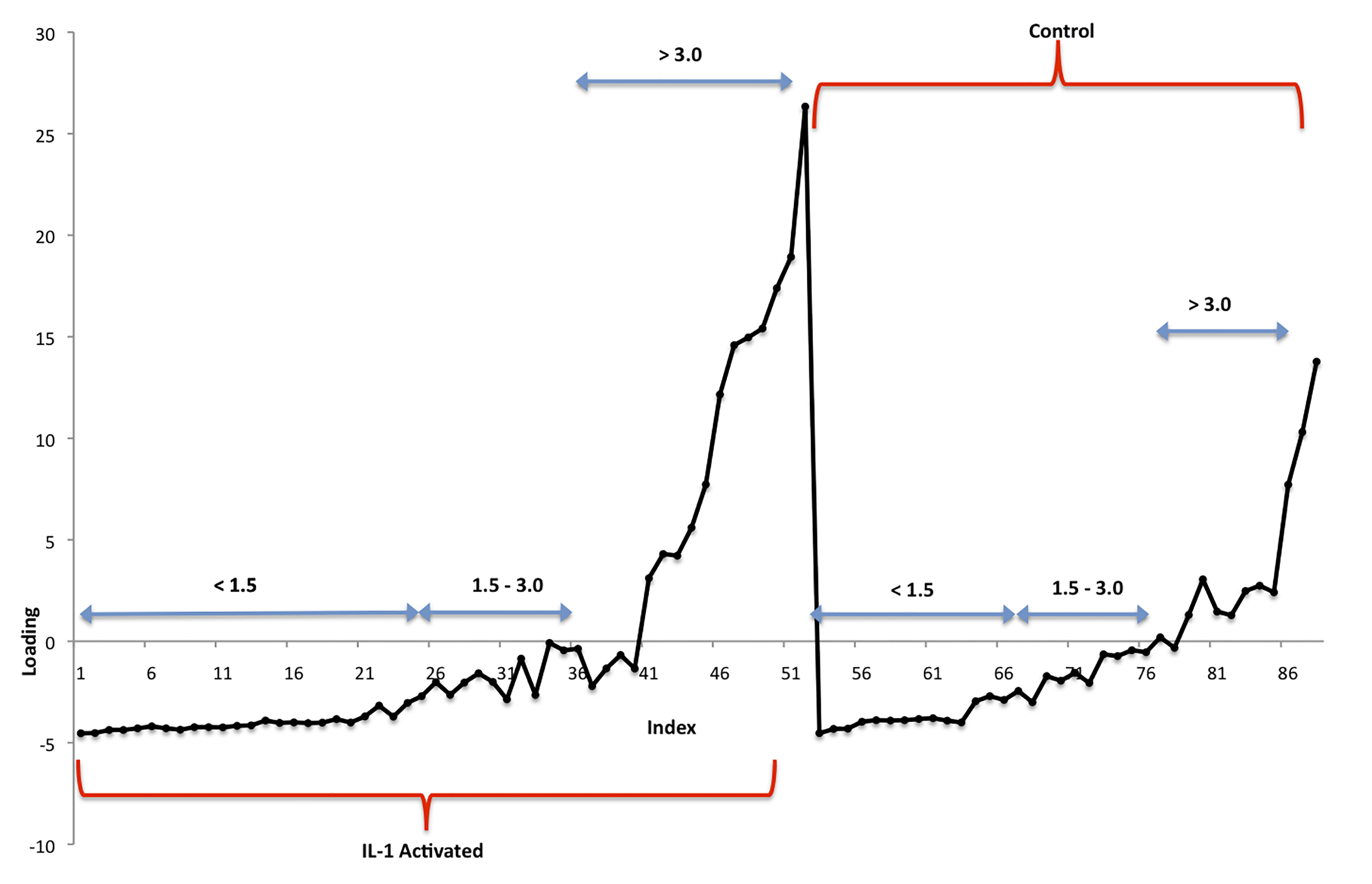

Supplement: S4 Fig — Plot of loadings for principal component 1 following PCA. PC1 was chosen because this is the component which contributes most to separation of the data. It can be seen that observations with initial fluorescence between 0-3.0 and >3.0 can be separated easily as the observation between 0-3.0 units have negative loadings and >3.0 have positive loadings. Furthermore, observations for cells with initial fluorescence between 0-1.5 tend to have relatively stable loadings (around -4.5), whereas those between 1.5-3.0 begin to have more variable loadings. (TIF) [file pone.0160834.s004.tif]
