# Supplementary material for: Statistical Techniques Complement UML When Developing Domain Models of Complex Dynamical Biosystems
Source: PLoS One. 2016 Aug 29;11(8):e0160834. doi: 10.1371/journal.pone.0160834 (PMC5003378; doi:10.1371/journal.pone.0160834)
Supplement: S1 Table — Subset of control observations from the single-cell analysis of Yang et al [32] that were used within our data analysis. (PDF) [file pone.0160834.s005.pdf]

**Observation**	**0 min**	**10 min**	**30 min**	**60 min**
1	0.18395574	0.124481328	0.088520055	0.085753804
2	0.225864454	0.22835408	0.220193638	0.22406639
3	0.261825726	0.24868603	0.216459198	0.187136929
4	0.416182573	0.427385892	0.39253112	0.36417704
5	0.493360996	0.519502075	0.420608575	0.313416321
6	0.494744122	0.475518672	0.378838174	0.364591978
7	0.567081604	0.331950207	0.442600277	0.414937759
8	0.609958506	0.485477178	0.402489627	0.369294606
9	0.614107884	0.510373444	0.439834025	0.381742739
10	0.629322268	0.427385892	0.329183956	0.313969571
11	0.762102351	0.322268326	0.233748271	0.190871369
12	0.992116183	0.862655602	0.919502075	0.852697095
13	1.093775934	1.027385892	1.014522822	0.994605809
14	1.315352697	1.112033195	0.755186722	0.522821577
15	1.362655602	1.249792531	1.129460581	0.862240664
16	1.381742739	0.771784232	0.676348548	0.663900415
17	1.556431535	1.570539419	1.453941909	1.523236515
18	1.902604887	1.46692024	1.147648686	1.08725219
19	1.991701245	1.526970954	1.414937759	1.46473029
20	2.078838174	1.46473029	1.008298755	0.796680498
21	2.079253112	2.108713693	2.063900415	1.997925311
22	2.115767635	1.928630705	2.063070539	1.969709544
23	2.205394191	2.194605809	2.172614108	2.079253112
24	2.264315353	2.304564315	1.950622407	1.919917012
25	2.821576763	2.651452282	2.261410788	2.153526971
26	2.929460581	2.468879668	1.937759336	1.46473029
27	3.119917012	2.947302905	2.960995851	3.116182573
28	3.928077455	3.87275242	3.513139696	4.370677732
29	3.962655602	2.55186722	3.062240664	2.850622407
30	4.033195021	3.481327801	2.7593361	1.676348548
31	4.107883817	4.089626556	3.135684647	3.094190871
32	4.116182573	4.165975104	3.609958506	3.053941909
33	5.988934993	3.402489627	2.65560166	2.102351314
34	6.708160443	7.123098202	5.325034578	5.739972337
35	8.312586445	8.049792531	7.662517289	5.988934993
36	8.755186722	12.48962656	9.211618257	6.390041494
