# Supplementary material for: Statistical Techniques Complement UML When Developing Domain Models of Complex Dynamical Biosystems
Source: PLoS One. 2016 Aug 29;11(8):e0160834. doi: 10.1371/journal.pone.0160834 (PMC5003378; doi:10.1371/journal.pone.0160834)
Supplement: S2 Table — Subset of IL-1 stimulated observations from the single-cell analysis of Yang et al [32] that were used within our data analysis. (PDF) [file pone.0160834.s006.pdf]

Observation	0 min	10 min	30 min	60 min
1	0.278008299	0.157676349	0.012448133	0.001383126
2	0.285892116	0.133748271	0.029045643	0.027662517
3	0.334716459	0.291839557	0.12724758	0.019363762
4	0.408990318	0.156016598	0.126279391	0.08340249
5	0.429737206	0.334163209	0.114522822	0.058229599
6	0.439834025	0.323651452	0.215767635	0.157676349
7	0.492946058	0.219363762	0.132780083	0.087551867
8	0.495988935	0.230290456	0.029460581	0.034439834
9	0.502074689	0.211618257	0.157676349	0.182572614
10	0.518672199	0.394190871	0.091286307	0.033195021
11	0.539419087	0.345781466	0.099585062	0.030428769
12	0.557399723	0.359612725	0.150760719	0.110650069
13	0.585753804	0.348547718	0.174412172	0.116874136
14	0.597510373	0.473029046	0.337482711	0.302904564
15	0.669709544	0.448824343	0.196957123	0.14868603
16	0.67648686	0.610096819	0.113416321	0.088658368
17	0.697648686	0.52406639	0.151728907	0.046196404
18	0.788934993	0.352143845	0.214661134	0.122544952
19	0.865836791	0.652835408	0.269709544	0.019363762
20	0.908713693	0.477178423	0.053941909	0.037344398
21	0.926694329	0.539419087	0.387275242	0.235131397
22	1.037344398	0.899031812	0.650069156	0.580912863
23	1.391701245	0.458921162	0.094190871	0.089211618
24	1.506224066	1.020746888	0.531120332	0.331950207
25	1.654356846	1.212863071	0.839004149	0.341078838
26	1.705394191	1.369294606	1.348547718	1.045643154
27	1.754356846	1.189626556	0.766804979	0.470124481
28	1.842323651	1.44813278	1.153526971	0.962655602
29	1.880082988	1.804564315	1.356016598	1.300414938
30	2.024896266	1.796680498	0.929460581	0.68879668
31	2.074688797	1.236514523	0.265560166	0.112033195
32	2.244813278	1.804979253	1.883817427	1.875518672
33	2.24813278	1.393360996	0.304979253	0.153941909
34	2.614107884	2.261410788	2.203319502	2.286307054
35	2.757261411	2.512033195	2.139419087	1.129875519
36	2.87966805	2.294605809	1.821576763	1.730290456
37	3.073858921	1.229045643	0.347302905	0.30746888
38	3.175518672	1.925311203	1.18340249	0.402074689
39	3.667219917	1.948547718	2.147302905	0.226141079
40	3.929460581	1.443983402	0.975103734	0.298755187
41	4.38450899	3.858921162	3.969571231	3.513139696
42	5.269709544	4.854771784	4.343015214	3.56846473
43	5.546334716	4.273858921	3.886583679	4.204702628
44	6.473029046	5.61549101	5.329183956	3.125864454
45	6.569847856	6.002766252	6.01659751	6.417704011
46	8.57538036	8.538035961	8.077455048	8.686030429
47	9.751037344	9.793222683	9.557538036	9.637482711
48	10.47026279	9.800829876	9.865836791	9.312586445
49	10.95435685	9.405255878	9.811618257	10.20746888
50	12.48962656	11.38312586	10.67773167	9.626556017
51	14.05255878	12.0055325	11.17565698	9.972337483
52	16.33471646	15.80912863	15.08990318	14.93775934
