# Supplementary material for: Statistical Techniques Complement UML When Developing Domain Models of Complex Dynamical Biosystems
Source: PLoS One. 2016 Aug 29;11(8):e0160834. doi: 10.1371/journal.pone.0160834 (PMC5003378; doi:10.1371/journal.pone.0160834)
Supplement: S3 Table — χ2 test for control observations approximating to a negative binomial distribution. (PDF) [file pone.0160834.s007.pdf]

**Value**	**Observed (O_i)**	**Expected (E_i)**	**O_i-E_i**	**(O_i-E_i)$_2$ / E_i**
< 1	12	11.225772	0.774228	0.0533976
1 - 2	7	8.7013764	-1.7013764	0.3326694
2 - 3	7	5.917482	1.082518	0.1980311
3 - 4	3	3.8369432	-0.8367432	0.1824827
> 4	7	6.3186264	0.6813736	0.0734764
**Total**	**36**	**36**	**0**	**0.8400572**
