# Supplementary material for: Statistical Techniques Complement UML When Developing Domain Models of Complex Dynamical Biosystems
Source: PLoS One. 2016 Aug 29;11(8):e0160834. doi: 10.1371/journal.pone.0160834 (PMC5003378; doi:10.1371/journal.pone.0160834)
Supplement: S4 Table — χ2 test for IL-1 stimulated observations approximating to a negative binomial distribution. (PDF) [file pone.0160834.s008.pdf]

**Value**	**Observed (O_i)**	**Expected (E_i)**	**O_i-E_i**	**(O_i-E_i)$_2$ / E_i**
< 1	21	15.9764696	5.0235304	1.5795641
1 - 2	8	11.3233276	-3.3233276	0.9753764
2 - 3	7	7.8343252	-0.8343252	0.0888524
3 - 4	4	5.3764620	-1.3764620	0.3523967
4 - 5	1	3.6742108	-2.6742108	1.9463781
5 - 6	2	2.5050012	-0.5050012	0.1018068
6 - 7	2	1.7049656	0.2950344	0.0510540
7 - 8	0	1.1590748	-1.1590748	1.1590748
8 - 9	1	0.7872644	0.2127356	0.0574857
9 - 10	1	0.5343520	0.4656480	0.4057776
> 10	5	1.1245468	3.8754532	13.3557247
**Total**	**52**	**52**	**0**	**20.0734913**
