# Supplementary material for: Statistical Techniques Complement UML When Developing Domain Models of Complex Dynamical Biosystems
Source: PLoS One. 2016 Aug 29;11(8):e0160834. doi: 10.1371/journal.pone.0160834 (PMC5003378; doi:10.1371/journal.pone.0160834)
Supplement: S5 Table — Summary of principal component analysis of the single-cell fluorescence data, showing the standard deviation, proportion of variance and cumulative proportion of variance for each principal component. (PDF) [file pone.0160834.s009.pdf]

	**PC1**	**PC2**	**PC3**	**PC4**
Standard Deviation	6.241	0.65322	0.48826	0.21399
Proportion of Variance	0.982	0.01076	0.00601	0.00115
Cumulative Proportion	0.982	0.99283	0.99885	1.00000
