# Supplementary material for: Statistical Techniques Complement UML When Developing Domain Models of Complex Dynamical Biosystems
Source: PLoS One. 2016 Aug 29;11(8):e0160834. doi: 10.1371/journal.pone.0160834 (PMC5003378; doi:10.1371/journal.pone.0160834)
Supplement: S1 File — (PDF) [file pone.0160834.s010.pdf]

Subset of Single-Cell Observations used within our Domain Model

The numerical data in S1 and S2 Tables relates to the cytoplasmic fluorescence levels at times 0, 10, 30 and 60 min. The observation number relates to the ID of the cell during single-cell analysis.

S1 Table. Control observations for data analysis. Multiple subsets of control observations selected based on initial transfection levels determined by single-cell analysis (as described in Yang et al [32]) that were used within our data analysis. See the subsection on Modelling Dynamics for specific analysis, and specifically the histogram depicting its negative binomial distribution (Fig 5), the median average fluorescence (Fig 7) and the PCA plot (Fig 8) that merged the dataset with the subset of IL-1 stimulated observations in S2 Table.

S2 Table. IL-1 stimulated observations for data analysis. Multiple subsets of IL-1 stimulated observations selected based on initial transfection levels determined by single-cell analysis (as described in Yang et al [32]) that were used within our data analysis. See the subsection on Modelling Dynamics for specific analysis, and specifically the histogram depicting its negative binomial distribution (Fig 6), the median average fluorescence (Fig 7) and the PCA plot (Fig 8) that merged the dataset with the subset of control observations in S1 Table.
