# Supplementary material for: Statistical Techniques Complement UML When Developing Domain Models of Complex Dynamical Biosystems
Source: PLoS One. 2016 Aug 29;11(8):e0160834. doi: 10.1371/journal.pone.0160834 (PMC5003378; doi:10.1371/journal.pone.0160834)
Supplement: S2 File — (PDF) [file pone.0160834.s011.pdf]

Chi-squared (χ^2) goodness of fit

As discussed by Tijskens et al [44] and Elowitz et al [45], a degree of variance is inherent to all aspects of biology due to the underlying stochastic physiological events of individual cells. The single-cell fluorescence data of Yang et al [31, 32] encapsulates this stochasticity, and we feel that this should be explored further within the domain model. For predictive purposes, and indeed to indicate the types of statistical tests that should be used in the future (and which graphical representations are best suited), it is often desirable to understand the shape of the underlying distribution of the data. To determine the underlying distribution, it is common to fit the observed data to a theoretical distribution by comparing the frequencies observed in the data to the expected frequencies of the theoretical distribution.

Given two sets of data, we can test to ascertain whether they come from the same population distribution using a number of statistical techniques. The accepted test for differences between non-continuous (binned) distributions is the chi-square (χ^2) test. The single-cell analysis fluorescence data of Yang et al [32] contains 36 observations for *control* (unstimulated) dynamics and 52 observations for *IL-1 stimulated* dynamics, with fluorescence readings at times 0 min, 15 min, 30 min, and 60min. These observations can be grouped into regular intervals (binned) for fluoresecence at particular time points, and are therefore amenable to the two-tailed χ^2 goodness of fit test to ascertain whether the data (control and IL-1 stimulated) approximates to known mathematical distributions. We have used Chi-squared (χ^2) goodness of fit tests to ascertain that the single-cell analysis (IκBα degradation) fluorescence data (at time 0min) of [32] approximates to a *Negative Binomial* distribution, which we believe follows the usual patterns in biology of variation due to stochasticity [44, 46, 70].

The data contained measurements from single-cell analysis performed on 88 cells: 52 were transfected with IκBα Enhanced Green Fluorescent Protein (EGFP) and stimulated with IL-1; and 36 were transfected with IκBα-EGFP, but not stimulated with extracellular ligand, thus representing a *control* group. Single-cell analysis occurs on live cells, with the same set of cells being followed over time. All measurements within the data related to cytoplasmic fluorescence and were taken over a period of one hour, at intervals corresponding to 0, 10, 30 and 60 min. Fig 5 in the manuscript represents the control data with integer binning and a superimposed curve that follows the negative binomial distribution with median average of 1.947153 (calculated from the control data). Similarly, fig 6 represents the IL-1 stimulated data with integer binning and a superimposed curve that follows the negative binomial distribution with median average of 1.729876 (calculated from the IL-1 stimulated data). The transformed data associated with these calculations are reproduced within S3 and S4 Tables. The hypothesis $H1_0$ is that the control observations approximate to a negative binomial distribution. The ${\chi_4}^2$ threshold for 97.5% is 11.483, and as the χ^2 score for the data is 0.84, we accept $H1_0$. Similarly, the hypothesis $H2_0$ is that the IL-1 stimulated observations approximate to a negative binomial distribution. The ${\chi_{10}}^2$ threshold for 97.5% is 20.483, and as the χ^2 score for the data is 20.07, we accept $H2_0$.

S3 Table. χ^2 test for control observations. χ^2 test for control observations approximating to a negative binomial distribution.

S4 Table. χ^2 test for IL-1 stimulated observations. χ^2 test for IL-1 stimulated observations approximating to a negative binomial distribution.
