# Supplementary material for: Statistical Techniques Complement UML When Developing Domain Models of Complex Dynamical Biosystems
Source: PLoS One. 2016 Aug 29;11(8):e0160834. doi: 10.1371/journal.pone.0160834 (PMC5003378; doi:10.1371/journal.pone.0160834)
Supplement: S3 File — (PDF) [file pone.0160834.s012.pdf]

Hierarchical Cluster Analysis

The negative binomial distribution is an alternative to the Poisson distribution, and is especially useful for data over an unbounded positive range whose sample variance exceeds the sample mean. Due to the large variance inherent in a negative binomial distribution; plotting a time course graph of mean average cytoplasmic fluorescence with standard deviation or variance as error bars, may not provide an understanding of the heterogeneous nature of the single-cell observations. The wet-lab data suggests that there is a residual/basal level of IκBα degradation within the system, and that following IL-1 stimulation this degradation rate increases. However, as a first step to allow IκBα degradation to be compared within unstimulated and IL-1 stimulated environments, we believe that the fluorescence data should be transformed so that each observation (an individual cell) become its own control, by dividing the cells' fluorescence reading at various time-steps by its initial fluorescence reading (at time 0 min). This converts the fluorescence for time 0 min for each cell to 1.0 (arbitrary units), and standardises the ensuing degradation over a 1 hr period. Our transformation of the data so that each observation becomes its own control at time 0 min, is consistent with [46]. They advise that an adequate fit of data to the negative binomial distribution provides a justification for transformation of the data to stabilize the variance, as a preparatory step for further statistical analysis by other techniques. Fig 7 in the main manuscript is a graph of the control (unstimulated) and IL-1 stimulated data using a subset of data that had initial fluorescence upto and including 1.5 arbitrary fluorescence units using median average and variance bars for interquartile ranges (25th to 75th percentiles). It can be seen that good separation is gained at 30 min onwards, with a little overlap still apparent at 10 min. The rates of degradation are 0.366 fluorescence units per hour for control and 0.864 fluorescence units per hour for IL-1 stimulated.

An alternative view in analysing the *full* time-series data is to visualise the four time measurements for each of the 88 observations using a scatterplot matrix (not shown). The most striking feature is that the data does not separate very well into simple groupings by stimulation status, i.e. control versus IL-1 stimulated, although there may be small regions of clustering at the lower levels of cytoplasmic fluorescence. This suggests that the dataset is either not independent, and as such may have dependencies between the underlying components of the system, or that the inherent stochasticity within the signalling pathway has yielded variation within the fluorescence measurements that prevents conclusions to be drawn using simple univariate statistical analysis. Due to the poor separation gained using the scatterplot matrix, hierarchical cluster analysis was used so that data may be quickly visualised in an alternative way using a multivariate technique [47, 48]. The raw data was encoded so that the measurement category (IL-1 v No IL-1) and repetition number were used to create a single label for each observation, e.g. observation 1 was coded as IL1_1 and related to the first single-cell observation with IL-1 stimulation.

Hierarchical clustering was performed using seven different clustering algorithms (Ward, single, complete, average, McQuitty, median and centroid) to find similarities in the single-cell observations and group them together to assist us in understanding any relationships that might exist among them. The resulting dendrograms for each method were consistent, in that no clear clustering was evident to group control cells and to group those stimulated with IL-1. There were however a few areas where clustering may be evident, and therefore appropriate for further investigation to ascertain any natural groupings within the data. Of note are the three main clusters obtained by scaling the data and using the *complete* clustering method (S1 Fig), which uses an agglomerative algorithm (using pairwise observations) on the maximum distance matrix. The first cluster (left) groups IL-1 stimulated observations with an initial cytoplasmic fluorescence (at 0 min) less than 3 fluorescence units (IL1_1 to IL1_34) and control

observations with an initial cytoplasmic fluorescence less than 2.079 fluorescence units. There are a few anomalous observations (IL1_37 to IL1_40), but overall the cluster aligns with our expectations. The second cluster, groups control observations 21 to 24 (No IL1_21 to No IL1_24) with initial cytoplasmic fluorescence of 2.079 to 8.0 and IL-1 stimulated observations IL1_41 to IL1_45, along with IL1_32, IL1_35 and IL1_36, which may be anomalies. The final cluster (actually clusters 3 and 4, but cluster 3 only has a single observation - IL1_52) groups both IL-1 stimulated and control observations with initial cytoplasmic fluorescence greater than 8.0 fluorescence units.

Scaling the data, slightly decreases the correlation coefficient of the data (between the distance matrix and the cophonetic distance) from 0.9159 to 0.8982, but enhances the clustering of IL-1 stimulated observations with each other, and control observations with each other (i.e. IL-1 stimulated observations group together and control observations group together), within the three main clusters. We believe that this slight reduction in correlation may be due to an amplification of standard error within the observations, however the correlation score is still high, and therefore we believe that the resulting dendrogram may be deemed an appropriate summary of the data.

S1 Fig. Dendrogram representing the clustering of single-cell analysis observations. Dendrogram representing the clustering of observations from [31] by hierarchical cluster analysis using the complete(-linkage) method. The boxes indicate that hierarchical cluster analysis identifies the three forced clusters as observations having an initial cytoplasmic fluorescence less than 3.0, between 3.0 and 8.0, and above 8.0 fluorescence units.
