# Supplementary material for: Statistical Techniques Complement UML When Developing Domain Models of Complex Dynamical Biosystems
Source: PLoS One. 2016 Aug 29;11(8):e0160834. doi: 10.1371/journal.pone.0160834 (PMC5003378; doi:10.1371/journal.pone.0160834)
Supplement: S4 File — (PDF) [file pone.0160834.s013.pdf]

S4 Principal Component Analysis

The scatterplot matrix and hierarchical cluster analysis provide preliminary indications of groupings/relationships between the data, however as no clear clustering is evident, a more powerful technique is required. Principal Component Analysis (PCA) is an unsupervised multivariate technique, which determines new variables (inherent to the dataset) based on the direction of maximum variance [49]. As such, it can be used to reduce the dimensionality of data for detecting underlying structures [50]. Within our analysis we removed the leading column from S1 and S2 Tables that related to observation number, to ensure that only the numerical data relating to cytoplasmic fluorescence at times 0, 10, 30, and 60 min were left.

The scree plot (S2 Fig) shows the variance corresponding to each *principal component* within the data of Yang et al [32]. It flattens out after principal component 1 (PC1), indicating that only a single principal component is required for the variation seen in the data. The cumulative proportions of variance (S5 Table) reinforce this hypothesis.

S2 Fig. Scree plot of the principal components from principal component analysis of the single-cell fluorescence data. Scree plot of the principal components from principal component analysis of observations from Yang et al [32]. Each bar corresponds to its respective principal component; bar heights are the variances of the principal components.

S5 Table. Summary of principal component analysis of the single-cell fluorescence data.

	**PC1**	**PC2**	**PC3**	**PC4**
Standard Deviation	6.241	0.65322	0.48826	0.21399
Proportion of Variance	0.982	0.01076	0.00601	0.00115
Cumulative Proportion	0.982	0.99283	0.99885	1.00000

Summary of principal component analysis of the single-cell fluorescence data, showing the standard deviation, proportion of variance and cumulative proportion of variance for each principal component.

A bi-plot of PC1 and PC2 provides another visual representation of these principal component loadings. S3 Fig shows that all four time measurements contribute to the separation of the data (through PC1), with times 0, 10 and 30 min having the greatest effect. Furthermore, it was found that: PC1 was dominated by measurements at times 0, 10 and 30 min; PC2 was dominated by measurements at times 0 and 30 min; PC3 was dominated by measurements at times 0 and 60 min; and PC4 was dominated by measurements at times 0 and 10 min.

S3 Fig. Bi-plot of PC1 and PC2 from principal component analysis of the single-cell fluorescence data. Bi-plot of PC1 and PC2 from principal component analysis of observations from Yang et al [32]. This plot shows that measurements for times 0, 10 and 30 min contribute equally to the separation of PC1 due to their virtually equivalent arrow lengths. They are not fully parallel to the PC1 axis however, and therefore also contribute slightly to PC2.

Subsequent analysis was performed on the four principal components, with the individual observations being coded depending on the relevant category. Initial

comparisons representing *control* and *IL-1 stimulated* observations did not yield separation of observations. Further analysis compared control and IL-1 stimulated conditions against the cytoplasmic fluorescence data expressed relative to levels at $time_0$. The best separation occurred using initial fluorescence ranges of 0-1.5 fluorescence units, consistent with the biological analysis by Carlotti et al [29] and Yang et al [31, 32]. Complete separation does not occur for any combination, however separation emerges between control and stimulated conditions for cells with initial cytoplasmic fluorescence up to 1.5 fluorescence units. As previously discussed in the main text of the manuscript, fig 8 represents the plot of PC1 versus PC2, which has been colour-coded to categorise control and IL-1 stimulated observations that have been grouped by their initial cytoplasmic fluorescence. There is limited separation between control and IL-1 stimulated cells with initial fluorescence levels of 1.5-3.0 and no appreciable difference at initial fluorescence > 3.0 units using PCA. Evidence for the separation of control and IL-1 stimulated observations with initial cytoplasmic fluorescence upto 3.0 fluorescence units is further provided through the plot of PC1 loadings against observation number (S4 Fig). This plot of loadings also confirms the separation of observations with initial cytoplasmic fluorescence upto 1.5 fluorescence units.

S4 Fig. Plot of loadings for PC1 following principal component analysis. Plot of loadings for principal component 1 following PCA. PC1 was chosen because this is the component that contributes most to separation of the data. It can be seen that observations with initial fluorescence between 0-3.0 and >3.0 can be separated easily as the observation between 0-3.0 units have negative loadings and >3.0 have positive loadings. Furthermore, observations for cells with initial fluorescence between 0-1.5 tend to have relatively stable loadings (around -4.5), whereas those between 1.5-3.0 begin to have more variable loadings.
